# A Genetic Toolbox for the New Model Cyanobacterium *Cyanothece* PCC 7425: A Case Study for the Photosynthetic Production of Limonene

**DOI:** 10.3389/fmicb.2020.586601

**Published:** 2020-09-18

**Authors:** Célia Chenebault, Encarnación Diaz-Santos, Xavier Kammerscheit, Sigrid Görgen, Cristian Ilioaia, Simona Streckaite, Andrew Gall, Bruno Robert, Elodie Marcon, David-Alexandre Buisson, Karim Benzerara, Jean-François Sassi, Corinne Cassier-Chauvat, Franck Chauvat

**Affiliations:** ^1^Université Paris-Saclay, CEA, CNRS, Institute for Integrative Biology of the Cell (I2BC), Gif-sur-Yvette, France; ^2^Sorbonne Université, Muséum National d’Histoire Naturelle, UMR CNRS 7590, Institut de Minéralogie, de Physique des Matériaux et de Cosmochimie, IMPMC, Paris, France; ^3^Université Paris-Saclay, Service de Chimie Bio-organique et Marquage (SCBM), CEA/DRF/JOLIOT, Gif-sur-Yvette, France; ^4^Commissariat à l’Énergie Atomique et aux Énergies Alternatives (CEA), Centre de Cadarache, Saint-Paul-lez-Durance, France

**Keywords:** conjugation, RSF1010 derivative plasmids, promoter probe vector, temperature-controlled expression vector, sub-cellular localization, growth on urea, terpene production

## Abstract

Cyanobacteria, the largest phylum of prokaryotes, perform oxygenic photosynthesis and are regarded as the ancestors of the plant chloroplast and the purveyors of the oxygen and biomass that shaped the biosphere. Nowadays, cyanobacteria are attracting a growing interest in being able to use solar energy, H_2_O, CO_2_ and minerals to produce biotechnologically interesting chemicals. This often requires the introduction and expression of heterologous genes encoding the enzymes that are not present in natural cyanobacteria. However, only a handful of model strains with a well-established genetic system are being studied so far, leaving the vast biodiversity of cyanobacteria poorly understood and exploited. In this study, we focused on the robust unicellular cyanobacterium *Cyanothece* PCC 7425 that has many interesting attributes, such as large cell size; capacity to fix atmospheric nitrogen (under anaerobiosis) and to grow not only on nitrate but also on urea (a frequent pollutant) as the sole nitrogen source; capacity to form CO_2_-sequestrating intracellular calcium carbonate granules and to produce various biotechnologically interesting products. We demonstrate for the first time that RSF1010-derived plasmid vectors can be used for promoter analysis, as well as constitutive or temperature-controlled overproduction of proteins and analysis of their sub-cellular localization in *Cyanothece* PCC 7425. These findings are important because no gene manipulation system had been developed for *Cyanothece* PCC 7425, yet, handicapping its potential to serve as a model host. Furthermore, using this toolbox, we engineered *Cyanothece* PCC 7425 to produce the high-value terpene, limonene which has applications in biofuels, bioplastics, cosmetics, food and pharmaceutical industries. This is the first report of the engineering of a *Cyanothece* strain for the production of a chemical and the first demonstration that terpene can be produced by an engineered cyanobacterium growing on urea as the sole nitrogen source.

## Introduction

Cyanobacteria, the oldest and largest phylum of prokaryotes that perform the plant-like photosynthesis (Schirrmeister et al., 2015), are regarded as the ancestors of the plant chloroplast (Ponce-Toledo et al., 2017) and the purveyors of the oxygen that shaped our biosphere (Hamilton et al., 2016). Contemporary cyanobacteria still capture a vast quantity of solar energy to assimilate huge amounts of CO_2_ (Dai et al., 2018) and nitrogen (nitrate, ammonium or urea) (Singh et al., 2016; Veaudor et al., 2019) to produce an enormous biomass that sustain most life forms on our planet. In colonizing most waters (fresh, brackish and marine) and soils (even deserts) biotopes, cyanobacteria have developed as widely diverse organisms. Their genomes vary in size (1.44–12.07 Mb) and organization (presence/absence of plasmids and linear chromosomes in addition to their circular chromosome) (Cassier-Chauvat et al., 2016). Furthermore, cyanobacteria display different cell sizes (1–10 μm) and morphologies, ranging from unicellular (cylindrical or spherical) (Koksharova and Wolk, 2002; Mazouni et al., 2004) to complex multi-cellular (filamentous) species (Cassier-Chauvat and Chauvat, 2014; Montgomery, 2015) capable to differentiate specialized cells for nitrogen fixation (Herrero et al., 2016) or survival to harsh environments (Chauvat et al., 1982; Legrand et al., 2019). Thus, cyanobacteria are interesting models to study how cells divide and pass their morphology on to their progeny (Cassier-Chauvat and Chauvat, 2014). Collectively, the disparities of the genome size, cell morphology and metabolism of cyanobacteria should prompt us to analyze a larger number of evolutionary-distant models to better understand and distinguish the common and species-specific aspects of cyanobacteria (Cassier-Chauvat and Chauvat, 2018).

Besides their great interest for basic science (Cassier-Chauvat and Chauvat, 2018), cyanobacteria are also regarded as promising cell factories for the production of chemicals for human health (Cassier-Chauvat et al., 2017; Demay et al., 2019) and industries (Cassier-Chauvat and Chauvat, 2018; Knoot et al., 2018). They capture solar energy at high efficiencies (3–9%) (Ducat et al., 2011) to fix a huge amount of carbon from atmospheric CO_2_ (about 25 gigatons annually) into a huge energy-dense biomass (Jansson and Northen, 2010), and they tolerate high CO_2_-containing (≥50%) industrial gas (Ducat et al., 2011). So far, only a handful of model strains with a well-established genetic have been engineered, such as *Synechocystis* PCC 6803, *Synechococcus* PCC 7942, or *Synechococcus* PCC 7002, leading to a weak and often transient production (Knoot et al., 2018; Lin and Pakrasi, 2019). Thus, the influence of the large biodiversity of cyanobacteria on the efficiency of the photosynthetic production of chemicals, has been overlooked.

For all the above-mentioned reasons we think that the time has come to enlarge the panel of the model cyanobacteria to better study and exploit their biodiversity for basic and applied research purposes.

In this study, we focused our attention on the poorly studied unicellular cyanobacterium *Cyanothece* PCC 7425 (also designated as *Cyanothece* ATCC 29141), which was isolated in 1972 from a rice paddy in Senegal (Rippka et al., 1979), because it has numerous attractive properties. *Cyanothece P*CC 7425 has larger cells (about 3–4 μm) (Porta et al., 2000; Bandyopadhyay et al., 2011) than the well-studied models *Synechococcus* PCC 7942 (cylindrical shape, 1.5 μm × 0.5 μm, Koksharova and Wolk, 2002) and *Synechocystis* PCC 6803 (1.5 μm in diameter, Mazouni et al., 2004). This feature should facilitate the analysis of the localization of proteins involved in assembly and distribution of the CO_2_-fixing carboxysomes and cell division, which are so far mainly studied in *Synechocystis* PCC 6803 and *Synechococcus* PCC 7942 (Cassier-Chauvat and Chauvat, 2014; Sommer et al., 2019; Sun et al., 2019; MacCready et al., 2020). Unlike these models, *Cyanothece* PCC 7425 can fix atmospheric nitrogen in anaerobiosis (Bandyopadhyay et al., 2011). It is also able to form intracellular CO_2_-sequestrating calcium carbonate granules, an interesting but as yet poorly studied particularity (Blondeau et al., 2018). *Cyanothece* PCC 7425 can also synthesize various biotechnologically interesting products, such as: (i) cyanophycin, the nitrogen-rich polymer of arginine and aspartate (Klemke et al., 2016); (ii) cyanobactins, a family of cyclic peptides (Houssen et al., 2012); (iii) alkane, sucrose and polyhydroxyalkanoates (biodegradable bioplastics) (Porta et al., 2000; Bandyopadhyay et al., 2011).

Furthermore, the genome of *Cyanothece* PCC 7425 (5.82 Mb) in being much larger than those of the well-studied models *Synechocystis* PCC 6803 (3.95 Mb), *Synechococcus* PCC 7002 (3.40 Mb) and *Synechococcus* PCC 7942 (2.75 Mb), should teach us new lessons about cyanobacteria. For example, *Cyanothece* PCC 7425 has the genes encoding the two (anti-oxidant) super-oxide dismutases SodA (Mn-dependent) and SodB (Fe-dependent), whereas *Synechocystis* PCC 6803, *Synechococcus* PCC 7002 and *Synechococcus* PCC 7942 only have SodB (Veaudor et al., 2019). Similarly, *Cyanothece* PCC 7425 encodes the anti-oxidant glutathione reductase enzyme that is missing in both *Synechocystis* PCC 6803 (Marteyn et al., 2009) and *Synechococcus* PCC 7002 (Narainsamy et al., 2013). *Cyanothece* PCC 7425 has two *radA* DNA-repair genes whereas all three model cyanobacteria have a single-copy *radA* gene (Cassier-Chauvat et al., 2016). Also interestingly, *Cyanothece* PCC 7425 possesses the full panoply of genes coding for urea uptake and catabolism, whereas *Synechococcus* PCC 7942 has no such genes, and *Synechocystis* PCC 6803 and *Synechococcus* PCC 7002 have no genes for the ureolytic enzymes urea carboxylase and allophanate hydrolase (Veaudor et al., 2019).

So far, only two attempts to manipulate a *Cyanothece* strain have been reported. First, a single-stranded spectinomycin resistance DNA-cassette, which could be introduced (by electro-transformation) and integrated into the genome of *Cyanothece* PCC 7822 cells, yielded no spectinomycin resistant clones when tested with *Cyanothece* PCC 7425 (Min and Sherman, 2010). More recently, Liberton et al. (2019) using DNA methylases to protect the incoming DNA from the *Cyanothece* ATCC 51142 restriction enzymes, could insert a kanamycin resistance cassette into the glycogen-catabolism gene glgX, generating a glycogen-rich mutant. However, this technique was not tested with *Cyanothece* PCC 7425. Thus, no gene manipulation system is yet available for *Cyanothece* PCC 7425, hampering its otherwise interesting potential to serve as a model host.

In this work, we first improved the classical BG-11 mineral medium cyanobacteria (Stanier et al., 1971) for better growth of *Cyanothece* PCC 7425, which appeared capable to grow not only on nitrate, the usual nitrogen source for cyanobacteria cultivated in the laboratory, but also on urea a frequent pollutant. Then, we developed a simple and efficient protocol for the conjugative transfer to *Cyanothece* PCC 7425 of the plasmid vectors derived from the broad-host-range plasmid RSF1010 that we previously constructed for gene manipulation in *Synechocystis* PCC 6803 and *Synechococcus* PCC 7942 (Marraccini et al., 1993; Mermet-Bouvier and Chauvat, 1994; Mazouni et al., 2004). We showed that these vectors replicate autonomously in *Cyanothece* PCC 7425 where they can be used for facile (i) promoter analysis, (ii) high-level, constitutive or temperature-controlled, protein productions, and (iii) analysis of sub-cellular localization of proteins. Finally, using this genetic toolbox we engineered a *Cyanothece* PCC 7425 strain for the stable photosynthetic production of limonene. This high-value terpene serves in cosmetics and food industries (Jongedijk et al., 2016), and it can be used as a fuel additive (Tracy et al., 2009; Chuck and Donnelly, 2014). This is the first report of the engineering of a *Cyanothece* strain for the photosynthetic production of a chemical, and the first demonstration that a terpene can be produced by an engineered cyanobacterium growing on urea as the sole nitrogen source. This suggests that it could be important in the future to couple chemicals productions with water treatment to reduce the costs (Veaudor et al., 2019).

## Results and Discussion

### Identification of Effective Conditions for the Growth of *Cyanothece* PCC 7425: Positive Influence of Calcium and Bicarbonate

**FIGURE 1:**
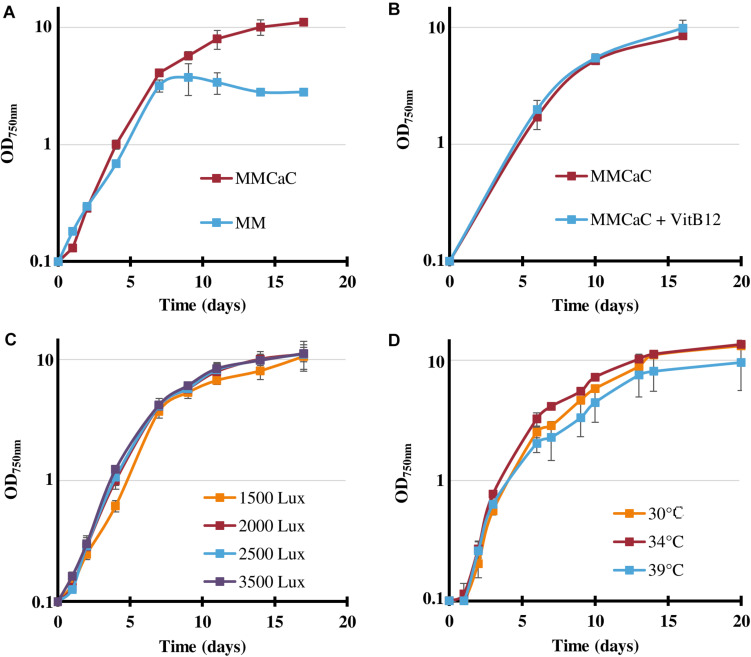
Influence of various conditions on the growth of *Cyanothece* PCC 7425. **(A)** Typical photoautotrophic growth of *Cyanothece* PCC 7425 at 30°C, 2000 lux (25.0 μE.m^–2^.s^–1^) in liquid mineral medium (MM) or MM supplemented with 9.52 mM NaHCO_3_ and 2.92 mM CaCl_2_ (MM_CaC_). **(B)** Influence of the addition of vitamin B12 (4 μg.L^–1^). **(C)** Influence of various light intensities on cell growth at 30°C in liquid MM_CaC_. **(D)** Influence of various temperatures on cell growth in liquid MM_CaC_ under 2000 lux. Error bars represent standard deviation from three biological replicates.

As *Cyanothece* PCC 7425 has been poorly studied so far, we first analyzed the influence of usually important parameters, such as light fluence, temperature and mineral availability, on its photoautotrophic growth, *Cyanothece* PCC 7425 appeared to grow well (Figure 1A) under the conditions defined as standard for *Synechocystis* PCC 6803, e.g., under white light 2000 lux (25.0 μE.m^–2^.s^–1^), at 30°C, in the MM mineral medium (Domain et al., 2004). As *Cyanothece* PCC 7425 possesses the full panoply of genes encoding the uptake and catabolism of urea that frequently occurs in natural waters (Veaudor et al., 2019), we have tested its capability to grow on urea as the sole nitrogen source. The results showed that cells grew well on urea up to 2 mM, whereas higher urea concentrations reduced the duration of healthy growth and production of biomass (Supplementary Figure S1). After 7–10 days of cultivation on urea, *Cyanothece* PCC 7425 can turn yellowish, as previously observed in the phylogenetically distant cyanobacteria *Anabaena cylindrica*, *Synechococcus* PCC 7002 (Sakamoto et al., 1998) and *Synechocystis* PCC 6803 growing on urea as the sole nitrogen source (Veaudor et al., 2019). Again as observed in *Synechocystis* PCC 6803 (Veaudor et al., 2018), once installed the chlorosis process decreased the cell viability measured by plating assays on standard growth medium (it contains nitrate, not urea). Interestingly, *Cyanothece* PCC 7425 grew up to increasing cell densities in response to increasing urea quantities, which needed to be supplied not all at once, but as small successive sub-doses along cell growth (Supplementary Figure S1). Also interestingly, the maximal growth (biomass production) of *Cyanothece* PCC 7425 was greatly improved by supplementing the MM with both 9.52 mM NaHCO_3_ and 2.92 mM CaCl_2_ (Figure 1A), in agreement with the previous finding that *Cyanothece* PCC 7425 forms intracellular calcium carbonate granules (De Wever et al., 2019).

Using the presently improved growth medium, hereafter designated as MM_CaC_, we found that the growth of *Cyanothece* PCC 7425 (doubling time about 24 h) is (i) similar under light intensities ranging from 1500 to 3500 lux (18.75–43.75 μE.m^–2^.s^–1^) (Figure 1C); (ii) not stimulated by the addition of vitamin B12 that can be beneficial or essential to cyanobacterial life (Figure 1B); and (iii) slightly improved or decreased by increasing the temperature to 34 or 39°C, respectively (Figure 1D).

### Development of an Effective Protocol for the Conjugative Transfer of RSF1010-Derived Replicative Plasmids to *Cyanothece* PCC 7425

Three decades ago, we contributed to the development of the genetics of the cyanobacteria *Synechocystis* PCC 6803 and *Synechococcus* PCC 7942, in using the broad-host-range plasmid RSF1010 for the construction of pSB2A, the first promoter-probe vector (Marraccini et al., 1993), and pFC1, the first conditional expression vector (Mermet-Bouvier and Chauvat, 1994). These vectors could be transferred by conjugation (or electroporation) from *Escherichia coli*, to these model cyanobacteria, where they stably replicate autonomously, though they contain no cyanobacterial origin of DNA replication (Mermet-Bouvier et al., 1993). Like RSF1010, pSB2A and pFC1 are not self-transmissible. They must be first introduced (by standard transformation) into an *E. coli* strain that already contains the self-transmissible RP4 plasmid, which cannot replicate in cyanobacteria, but encodes in *trans* the functions promoting the transfer of pSB2A or pFC1 to cyanobacteria. The resulting *E. coli* donor cells possessing both RP4 and pSB2A, or pFC1, are co-incubated with the cyanobacterial recipient cells in liquid medium. Then, the mixture is plated on solid mineral medium for selecting the cyanobacterial conjugants based on their antibiotic resistance encoded by pSB2A or pFC1 (Mermet-Bouvier et al., 1993).

In the present study, the pSB2A and pFC1 plasmids were used to test whether RSF1010-derivatives can be transferred by conjugation from *E. coli* to *Cyanothece* PCC 7425, and whether they can stably replicate autonomously in this cyanobacterium as observed in *Synechocystis* PCC 6803 and *Synechococcus* PCC 7942 (Mermet-Bouvier et al., 1993). For this purpose, a simpler and faster efficient protocol was developed that bypassed the introduction of pSB2A or pFC1 into the RP4-harboring *E. coli* strain that normally precedes and enables their conjugative transfer to cyanobacteria. Instead, the cyanobacterial recipient strain was co-incubated, on plate (not in liquid medium) in order to favor cell-cell interaction, with the two *E. coli* strains, one harboring RP4 and the other one pSB2A or pFC1. Also interestingly, similar high frequencies of conjugation, about 5.10^–4^ per cyanobacterial cell, were obtained when pSB2A or pFC1 were propagated in commonly used (*recA* KO) strains of *E. coli*, such as MC1061, TOP10 or XL1-Blue.

### The RSF1010-Derived Plasmid Vector pSB2A Can Serve for Promoter Analysis in *Cyanothece* PCC 7425

In this section we used pSB2A, the first promoter-probe-vector originally developed for *Synechocystis* PCC 6803 and *Synechococcus* PCC 7942 (Marraccini et al., 1993; Dutheil et al., 2012). pSB2A possesses a multiple cloning site for cloning any studied promoter in front of the promoter-less chloramphenicol acetyl transferase (*cat*) reporter gene. When expressed, for example by the strong *E. coli tac* promoter we cloned in pSB2A, yielding pSB2T (Supplementary Table S1), *cat* directs the production of the CAT reporter enzyme. The activity of this enzyme can be easily monitored by a spectrophotometric assay (Ferino and Chauvat, 1989) and confers the resistance to chloramphenicol (Marraccini et al., 1993; Dutheil et al., 2012) and references therein.

**FIGURE 2:**
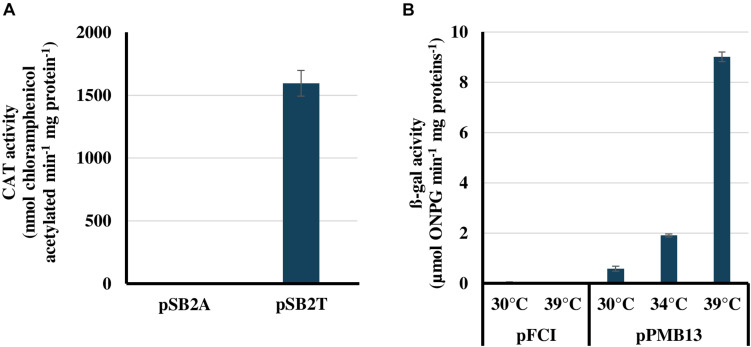
Validation of the pSB2A and pFC1 plasmid vectors for promoter analysis and temperature-controlled protein production in *Cyanothece* PCC 7425, respectively. **(A)** Chloramphenicol acyl transferase (CAT) activities of *Cyanothece* PCC 7425 cells propagating either the promoter probe vector pSB2A, which harbors the promoter-less *cat* reporter gene, or its pSB2T derivative, which expresses the *cat* gene from the strong *E. coli tac* promoter. **(B)** β-galactosidase activities of *Cyanothece* PCC 7425 cells propagating either the temperature-controlled expression vector pFC1 or its pMB13 derivative harboring the *lacZ* protein coding sequence. All activities are the mean values of three measurements performed on two different cellular extracts.

The presently reported conjugation protocol was used to introduce the Sm^R^/Sp^R^ plasmids pSB2A and pSB2T in *Cyanothece* PCC 7425. Among the large number of Sm^R^/Sp^R^ conjugants obtained, two independent clones were collected, re-streaked on Sm^R^/Sp^R^ plates and analyzed by PCR using relevant oligonucleotides primers (Supplementary Table S2). The results showed that pSB2A and pSB2T replicate stably in *Cyanothece* PCC 7425 (Supplementary Figure S2). Then, we measured the *cat* activities of the *Cyanothece* PCC 7425 reporter strains propagating pSB2A or pSB2T. As expected, a strong CAT activity was observed in *Cyanothece* PCC 7425 cells propagating pSB2T (Figure 2A). In contrast, no CAT activities were detected in *Cyanothece* PCC 7425 WT cells, which have no *cat* gene, and pSB2A-propagating cells, where the *cat* reporter gene is not expressed. Collectively, these results showed that the replicative promoter-probe vector pSB2A can be used for promoter analyses in *Cyanothece* PCC 7425.

### The RSF1010-Derived Plasmid Vector pFC1 Can Be Used for Temperature-Controlled Protein Production in *Cyanothece* PCC 7425

In this section, we used pFC1, the first conditional expression vector for cyanobacteria, originally developed for *Synechocystis* PCC 6803 and *Synechococcus* PCC 7942 (Mermet-Bouvier and Chauvat, 1994). pFC1 harbors the lambda-phage *cI*_857_ gene encoding the temperature-sensitive repressor that tightly controls the activity of the otherwise strong *p*_R_ promoter located behind *cI*_857_ (in opposite direction) (Mermet-Bouvier and Chauvat, 1994). The *p*_R_ promoter is followed by a canonical ribosome-binding site (RBS, 5′-AGGA-3′) and a correctly spaced ATG initiation codon embedded within the unique *Nde*I restriction site (5′-CAT**ATG**-3′) for easy cloning and strong conditional expression of the studied protein-coding sequences (Sakr et al., 2013; Ortega-Ramos et al., 2014) and references therein.

The Sm^R^/Sp^R^ plasmids pFC1, and its pPMB13 derivative for temperature-controlled expression of the *E. coli lacZ* gene encoding the beta-galactosidase reporter enzyme (Mermet-Bouvier and Chauvat, 1994), were introduced by conjugation (see above) in *Cyanothece* PCC 7425. Among the large number of Sm^R^/Sp^R^ conjugant clones obtained, two independent clones were collected and re-streaked on Sm^R^/Sp^R^ plates, prior to PCR analyses that showed that pFC1 and pPMB13 replicate stably in *Cyanothece* PCC 7425 (Supplementary Figure S3). Then, we measured the beta-galactosidase activities of the *Cyanothece* PCC 7425 reporter strains propagating pFC1 or pPMB13. No beta-galactosidase activities were detected in cells propagating pFC1, which lacks *lacZ*. A weak beta-galactosidase activity was observed in cells propagating pPMB13 grown at 30°C where *lacZ* expression is mostly blocked by the temperature-sensitive repressor CI_857_ produced by pPMB13. Finally, the transfer of pPMB13 reporter cells to higher temperatures increased *lacZ* expression, proportionally to the growth temperature, i.e., moderately at 34°C and massively at 39°C, as expected (Figure 2B). These results showed that the autonomously replicating plasmid vector pFC1 can be used for temperature-regulated protein production in *Cyanothece* PCC 7425, as was observed in *Synechocystis* PCC 6803 for many endogenous proteins (Sakr et al., 2013; Ortega-Ramos et al., 2014) and references therein. This system for tight control of (strong) gene expression is very interesting when one wants to produce chemicals that are toxic and prevent cell growth or generates mutations that decrease the production to escape cell death (Cassier-Chauvat et al., 2016). Using such a tightly controlled production system it is possible to first grow the engineered cyanobacterium up to a large cell population, before triggering the production of the toxic product which should thus be more efficient (Cassier-Chauvat et al., 2016).

### RSF1010-Derived Plasmids and the Green-Fluorescent Reporter Protein Can Be Used to Analyze Protein Localization in *Cyanothece* PCC 7425

In a few model cyanobacteria, such as *Synechocystis* PCC 6803 and *Synechococcus* PCC 7942, translational fusion of studied proteins to the green-fluorescent reporter protein (GFP) proved useful to analyze the sub-cellular localization of proteins involved in cell division (Cassier-Chauvat and Chauvat, 2014) or the biogenesis of the CO_2_-fixing carboxysome microcompartment (Cameron et al., 2013).

To test whether the GFP reporter protein can be employed to study protein localization in *Cyanothece* PCC 7425, we tried to conjugate it with our previously constructed plasmids pSB2T-ftsZ-gfp, pSB2T-gfp-ftn6 and pSB2T-gfp-sepF that produce the *Synechocystis* PCC 6803 cytokinetic proteins FtsZ, SepF, and Ftn6 translationally fused to GFP (Mazouni et al., 2004; Marbouty et al., 2009). All attempts were unsuccessful, suggesting that these fusion proteins impair the crucial cytokinetic process of *Cyanothece* PCC 7425.

Consequently, we tried to introduce in *Cyanothece* PCC 7425 the presently constructed pSB2T-derived plasmids pSB2T-ccmk1_tsbp__1_-gfp and pSB2T-maf_S__6803_-gfp which encode GFP fusions with the presumptive carboxysome protein CcmK1 (Tll0946) of the cyanobacterium *Thermosynechococcus elongatus* BP1 (Cameron et al., 2013) and the *Synechocystis* PCC 6803 presumptive cytokinetic protein (Sll0905) Maf (Hamoen, 2011), respectively (Supplementary Figure S2). pSB2T-ccmk1_tsbp__1_-gfp was constructed by cloning downstream of the *tac* promoter of pSB2T opened at its unique *Hpa*I restriction site, an *Eco*RV restriction fragment containing the *ccmk1*_tsbp__1_-*gfp* fusion gene (synthetized by Genecust; Supplementary Figure S4). Similarly, pSB2T-maf_S__6803_-gfp harbors the *maf*_S__6803_-*gfp* fusion gene downstream of its *tac* promoter (Supplementary Figures S2, S5).

**FIGURE 3:**
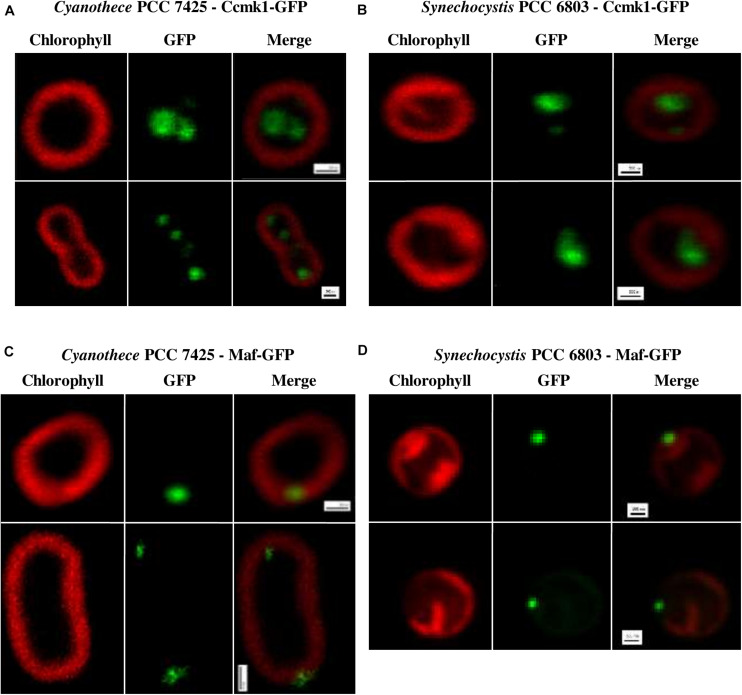
Localization of the fusion proteins Ccmk1_tsbp1_-GFP and Maf_S6803_-GFP in *Cyanothece* PCC 7425 and *Synechocystis* PCC 6803. Fluorescence images (scale bars, 500 nm) of *Cyanothece* PCC 7425 and *Synechocystis* PCC 6803 reporter cells propagating the plasmids pSB2T-ccmk1_tsbp1_-gfp **(A,B)** or pSB2T-maf_S6803_-gfp **(C,D)**, respectively.

These plasmids, along with the previously constructed pSB2T-GFP plasmid encoding the unfused GFP (Mazouni et al., 2004), were transferred by conjugation to *Cyanothece* PCC 7425 and also to *Synechocystis* PCC 6803 as a control strain. Similar high-numbers of conjugant clones were obtained in all cases. PCR analyses showed that all three plasmids appeared to propagate stably in *Synechocystis* PCC 6803 and *Cyanothece* PCC 7425 (Supplementary Figure S2). In both hosts, the GFP protein alone displayed no particular localization, as previously observed in *Synechocystis* PCC 6803 (Mazouni et al., 2004). In contrast, the CcmK1_tsbp__1_-GFP protein appeared to localize inside the cells of both *Synechocystis* PCC 6803 and *Cyanothece* PCC 7425 (Figures 3A,B), likely in their carboxysome as observed with the similar CcmK1_S__7942_-GFP fusion protein in *Synechococcus* PCC 7942 (Cameron et al., 2013). The localization of the Maf_S__6803_-GFP fusion protein was also similar in both *Synechocystis* PCC 6803 and *Cyanothece* PCC 7425, but this protein was accumulated in one to two spots at the inner periphery of the cells (Figures 3C,D), unlike the CcmK1_tsbp__1_-GFP protein. Collectively these data show that RSF1010-derived plasmids and the fluorescent GFP reporter protein are useful tools to study protein localization in the interesting cyanobacterium *Cyanothece* PCC 7425 that has larger cells than the models *Synechocystis* PCC 6803 and *Synechococcus* PCC 7942 classically used for analyzing the sub-cellular localization of proteins.

### Construction of a *Cyanothece* PCC 7425 Strain Carrying the *Mentha spicata* Limonene Synthase Encoding Transgene Expressed From the Strong Lambda-Phage p_R_ Promoter of a RSF1010-Derived pC Plasmid Vector

Then we tested whether *Cyanothece* PCC 7425 can be used for the photosynthetic production of high-value chemicals, such as limonene (C_10_H_16_). This volatile terpene, naturally produced by plants, has applications in biofuels (Tracy et al., 2009; Chuck and Donnelly, 2014), bioplastics, cosmetic and pharmaceutical industries (Jongedijk et al., 2016). We employed the limonene synthase from *Mentha spicata* because it efficiently transforms the geranyl diphosphate metabolite produced by the NAD(P)H-dependent MEP pathway, into limonene of high purity (Colby et al., 1993). Furthermore, this plant enzyme worked well in the model cyanobacteria *Synechococcus* PCC 7002 (Davies et al., 2014), *Synechococcus* PCC 7942 (Wang et al., 2016) and *Synechocystis* PCC 6803 (Lin et al., 2017). Like previous workers, we removed the first 168 bp encoding the chloroplast targeting sequence from the *Mentha spicata* limonene synthase gene (*ls*) and we adapted its coding sequence to the cyanobacterial usage of codon. We chose the codon usage of *Synechocystis* PCC 6803, because it is similar to that of *Cyanothece* PCC 7425 and proteins efficiently produced in *Synechocystis* PCC 6803 are also well produced in *Cyanothece* PCC 7425 (Figures 2, 3). The *ls* nucleotide sequence was synthesized by the Eurofins company as a DNA segment flanked by *Nde*I and *Eco*RI restriction sites at its 5′- and 3′-ends, respectively. After cleavage with both *Nde*I and *Eco*RI, the *ls* gene was cloned downstream of the strong λ*p*_R_ promoter of the pFC1-derivative pC plasmid for high-level constitutive gene expression (Veaudor et al., 2018), which was opened with the same enzymes (Supplementary Figures S6, S7).

**FIGURE 4:**
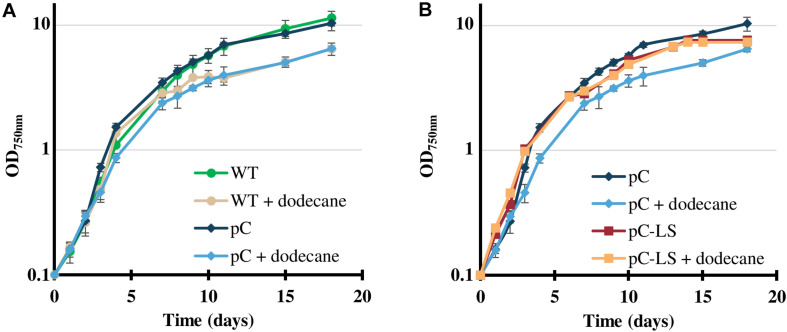
Influence of the pC-LS plasmid expressing the limonene synthase gene on the photoautotrophic growth of *Cyanothece* PCC 7425, in absence or presence of a dodecane overlay. **(A)** Typical photoautotrophic growth of the *Cyanothece* PCC 7425 wild-type strain (WT) and its derivative propagating the pC plasmid, at 30°C, 2000 lux (25.0 μE.m^–2^.s^–1^) in liquid MM_CaC_ mineral medium, in the presence or absence of a 20% (vol/vol) dodecane overlay. **(B)** Typical photoautotrophic growth in the same conditions of *Cyanothece* PCC 7425 strains propagating pC or its pC-LS derivative for high-level constitutive expression of the limonene synthase gene. Error bars represent standard deviation from three biological replicates.

The resulting pC-LS plasmid (Supplementary Figure S6) was transferred by conjugation to *Cyanothece* PCC 7425. Two independent Sm^R^/Sp^R^ clones were selected and analyzed by PCR and DNA sequencing (Supplementary Figure S3), which showed that pC-LS replicates stably in *Cyanothece* PCC 7425. As expected, the presence of the pC-LS plasmid did not adversely affect the photoautotrophic growth of *Cyanothece* PCC 7425 (Figures 4A,B), irrespectively of the number of sub-cultivation (regular verifications were performed during 12 months). Because of the volatility of limonene, a dodecane overlay was applied on cultures to collect limonene in the organic layer. As observed with *Synechococcus* PCC 7002 (Davies et al., 2014) and *Synechocystis* PCC 6803 (Lin et al., 2017), the dodecane overlay had little influence on the growth of *Cyanothece* PCC 7425 (Figures 4A,B), and the C12 chain length of dodecane (C_12_H_26_) allowed chromatographic separation from the C10 length of limonene (C_10_H_16_) (Supplementary Figure S8).

### The Engineered Strain of *Cyanothece* PCC 7425 Propagating the Limonene Synthase Expression Vector pC-LS Is a Stable Limonene Producer

**FIGURE 5:**
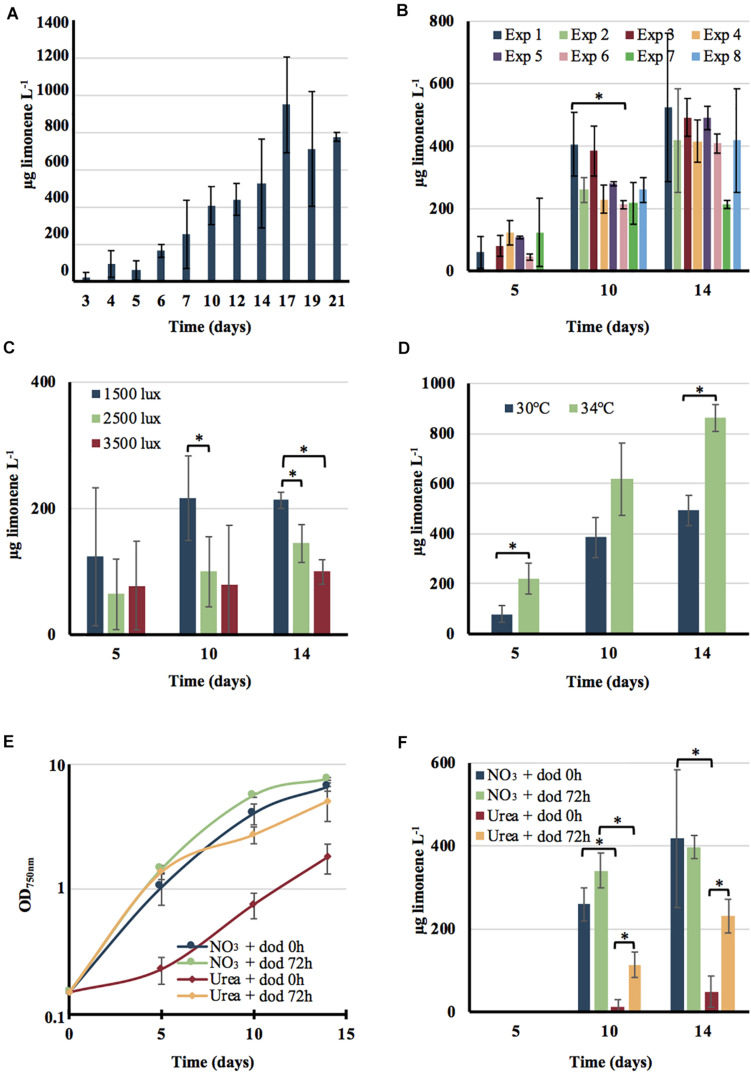
Influence of various growth conditions on the production of limonene by the engineered strain of *Cyanothece* PCC 7425. Cells growing in MM_CaC_ at 30°C under a 1500 lux light (18.75 μE.m^–2^.s^–1^) were inoculated at the initial cell density of 4 × 10^6^ cells mL^–1^ (OD_750 nm_ = 0.15) and further grown under the indicated conditions. Limonene production was monitored at the indicated time intervals. **(A)** Cells were grown for 21 days under above indicated conditions in the presence of a 20% (vol/vol) dodecane overlay. **(B)** Cells were sub-cultured in standard conditions (absence of dodecane) for variable periods (a few weeks to 9 months) prior to assay limonene production as described above during 14 days periods. **(C)** Cells were grown for 14 days in MM_CaC_ at 30°C under the indicated light intensities: 1500 lux (18.75 μE.m^–2^.s^–1^), 2500 lux (31.25 μE.m^–2^.s^–1^) or 3500 lux (42 μE.m^–2^.s^–1^) in the presence of a 20% (vol/vol) dodecane (dod) overlay prior to test limonene production. **(D)** Cells were grown at 30°C or 34°C in MM_CaC_ under a 1500 lux light (18.75 μE.m^–2^.s^–1^) and a 20% (vol/vol) dodecane overlay. Typical growth **(E)** and limonene production **(F)** of cells incubated at 30°C under 1500 lux (18.75 μE.m^–2^.s^–1^) in a nitrogen-free MM_CaC_ medium (MM_*CaC0N*_), which was supplemented at times 0, 4, and 8 days with the same amounts of nitrogen. They were provided as successive additions of either 2 mM nitrate (NO_3_; up to 8 mM final) or 1 mM urea [CO(NH_2_)_2_; up to 4 mM final]. The dodecane overlay was added either at the beginning of the experiment (dodecane 0 h) or after 72 h of growth. In all cases, error bars represent standard deviation from three biological replicates. The hooks ⊏ indicate a significant difference between the two compared experiments (*t*-test, *P* < 0.05; symbolized by *).

The rate of limonene biosynthesis during the growth phase of the *Cyanothece* PCC 7425 strains propagating the pC-LS plasmid or the (empty) pC vector, were measured over a 21-days period of cultures with a 20% (v/v) dodecane overlay (Figure 5A). GC-MS analysis of dodecane overlay samples from the pC-LS propagating strain showed a prominent peak with a similar retention time to pure commercial standards of *S*-(-)-limonene (6.90 min). This peak, which was not observed in the case of the negative control strain propagating the (empty) pC vector, displays a similar pattern than the *S*-(-)-limonene reference spectra from the NIST Mass Spectral Library (Supplementary Figure S8).

The overall limonene production, about 0.6–1.0 mg/L, was unaffected by the duration of sub-cultivation under photoautotrophic conditions (up to 9 months) of the engineered *Cyanothece* PCC 7425 strain, prior to limonene assay (Figures 5A,B). This test, rarely presented in articles reporting the engineering of cyanobacteria for chemical production (Knoot et al., 2018; Lin and Pakrasi, 2019), showed that the *Cyanothece* PCC 7425 limonene producer is genetically stable. This finding is interesting since it is known that cyanobacteria engineered for chemical production can be genetically unstable (for a review see Cassier-Chauvat et al., 2016). The level of limonene production by the engineered *Cyanothece* PCC 7425 strain was slightly increased by either decreasing the light down to 1500 lux or shifting the temperature up to 34°C (Figures 5C,D). In every cases it was similar to what was reported for *Synechococcus* PCC 7942 (Wang et al., 2016), but lower (about fivefold) than what was reported for the very-well known model cyanobacteria *Synechococcus* PCC 7002 (Davies et al., 2014) and *Synechocystis* PCC 6803 (Lin et al., 2017). However, it is very difficult to meaningfully compare limonene production from different cyanobacteria growing under various conditions in different laboratories. Future work will be required to attempt at increasing limonene production by the engineered *Cyanothece* PCC 7425. This will involve finding better conditions for cell growth and limonene collection, as well as effective metabolic engineering strategies.

### The Engineered Strain of *Cyanothece* PCC 7425 Can Photosynthetically Produce Limonene, Using Either Nitrate or Urea as the Sole Nitrogen Source

As we have shown above, *Cyanothece* PCC 7425 can grow not only on nitrate, the usual nitrogen source of cyanobacteria, but also on urea, a frequent and abundant pollutant (Veaudor et al., 2019). Growth on urea occurs when it is supplied not all at once, but as small successive sub-doses along cell growth (Supplementary Figure S1). Consequently, we compared the level of limonene production by our engineered strain of *Cyanothece* PCC 7425 growing either on nitrate or urea added in similar small quantities along cell growth. As compared to cells growing on nitrate, cells growing on urea can be affected by the dodecane overlay used for limonene trapping (Figure 5E). Both cell growth and limonene production are reduced when the dodecane overlay is applied at the beginning of the culture (when the cell density is very low) (Figures 5E,F). The negative influence of dodecane on urea-growing cells is reduced when it is applied after 72 h of growth (when the cell density is higher). Such urea-growing cells produced limonene up to about 50% of the quantity produced by cells growing on nitrate. Future work will be required to find good conditions for the photosynthetic production of limonene by *Cyanothece* PCC 7425 growing either on urea. This objective is important in the future view of using cyanobacteria for the photosynthetic production of chemicals coupled with water treatment to reduce the costs (Veaudor et al., 2019).

## Conclusion

We have developed a genetic toolbox for the robust unicellular cyanobacterium *Cyanothece* PCC 7425. We first showed that the addition of extra calcium and carbonate to the BG-11 mineral medium, classically used to grow cyanobacteria (Stanier et al., 1971), improved the growth of *Cyanothece* PCC 7425. Then, we showed that *Cyanothece* PCC 7425 can grow, not only on nitrate the standard nitrogen source of cyanobacteria, but also on urea a frequent and abundant pollutant (Veaudor et al., 2019). Then, we developed a simple and efficient protocol for the conjugative transfer to *Cyanothece* PCC 7425 of plasmid vectors derived from the broad-host-range plasmid RSF1010. We previously constructed these vectors for (i) promoter analysis (Marraccini et al., 1993), (ii) constitutive or temperature-controlled protein productions (Mermet-Bouvier and Chauvat, 1994), and (iii) analysis of sub-cellular localization of proteins (Mazouni et al., 2004; Marbouty et al., 2009) in the model cyanobacteria *Synechocystis* PCC 6803 and *Synechococcus* PCC 7942. As expected, these autonomously replicating plasmid vectors appeared to work well in *Cyanothece* PCC 7425.

To emphasize the interest of this genetic toolbox for gene manipulation in *Cyanothece* PCC 7425, we used it to engineer a strain for the photosynthetic production of the high-value chemical limonene. Limonene, a 10-carbons volatile terpene (C_10_H_16_) normally produced by plants, is commercially used in cosmetics and food industries (Jongedijk et al., 2016), and it can be used as an additive to diesel (Tracy et al., 2009) or jet-fuel (Chuck and Donnelly, 2014; Lin et al., 2017). Finally, we have also shown that our engineered strain of *Cyanothece* PCC 7425 is genetically stable and it can produce limonene in calcium-rich water using either nitrate or urea as the sole nitrogen source. This is the first report of the engineering of a *Cyanothece* strain for the photosynthetic production of a chemical. It is also the first demonstration that a terpene can be produced by an engineered cyanobacterium growing on urea as the sole nitrogen source. This finding is interesting, because it could be important in the future to couple chemicals productions with wastewater treatment to reduce the costs (Veaudor et al., 2019).

## Materials and Methods

### Bacterial Strains and Growth Condition

*Cyanothece* PCC 7425 and *Synechocystis* PCC 6803 were grown at 30°C, in the MM mineral medium that corresponds to BG-11 (Stanier et al., 1971) enriched with 3.78 mM Na_2_CO_3_ (Domain et al., 2004), under continuous agitation (140 rpm, Infors rotary shaker) and cool white light. The intensities were, respectively 1500–2000 lux (18.75–25.00 μE.m^–2^.s^–1^) for *Cyanothece* PCC 7425 and 2500 lux (31.25 μE.m^–2^.s^–1^) for *Synechocystis* PCC 6803. Both of them were also grown on MM solidified with 10% Bacto Agar (Difco). For some experiments performed with *Cyanothece* PCC 7425, MM was supplemented with 9.52 mM NaHCO_3_ and 2.92 mM CaCl_2_ (hereafter designated as MM_CaC_) and/or nitrate was replaced by urea (1–4 mM), as indicated. In these latter cases, nitrate-grown cells were washed twice with nitrate-free medium before resuspension in urea-containing medium.

*Escherichia coli* strains MC1061 (Casadaban and Cohen, 1980), TOP10 (Invitrogen) or XL1-Blue (Agilent) were used for gene manipulations and/or conjugative transfer (CM404, Mermet-Bouvier et al., 1993) of RSF1010-derived replicative plasmids to *Cyanothece* PCC 7425 and *Synechocystis* PCC 6803 (CM404) (Supplementary Table S1). They were grown on LB medium at 30°C (CM404 and all cells harboring pFC1 derivatives) or 37°C (all other strains). Antibiotic selections were as follows for *E. coli*: chloramphenicol (Cm) 34 μg.mL^–1^, kanamycin (Km) 50 μg.mL^–1^, streptomycin (Sm) 25 μg.mL^–1^ and spectinomycin (Sp) 75 μg.mL^–1^; for *Cyanothece* PCC 7425: Cm 10 μg.mL^–1^, Km 25 μg.mL^–1^, Sm 2 μg.mL^–1^, and Sp 10 μg.mL^–1^; for *Synechocystis* PCC 6803: Km 50 μg.mL^–1^, Sp 5 μg.mL^–1^, and Sm 5 μg.mL^–1^.

### Conjugative Transfer of RSF1010- Derived Plasmids to *Cyanothece* PCC 7425 or *Synechocystis* PCC 6803

All plasmids were transferred from *E. coli* to cyanobacterial cells by trans-conjugation (Mermet-Bouvier et al., 1993), using a simplified and more efficient procedure involving a triparental mating and the co-incubation of *E. coli* and recipient cyanobacterial cells on solid medium. Five mL overnight-grown cultures of *E. coli* CM404 harboring the self-transferable mobilization vector (pRK2013, a derivative of RP4, Mermet-Bouvier et al., 1993) and a 5 mL overnight grown culture of *E. coli* strains harboring one of the studied pSB2A- or pFC1-derivative plasmids were washed twice with LB and resuspended separately into 800 μL LB (final concentration of 1.3 × 10^9^ cells.mL^–1^). Hundred μL *Cyanothece* PCC 7425 mid-log phase culture (about 1.25 × 10^7^ cells) were mixed with 30 μL of a CM404 *E. coli* culture and 30 μL of a culture of an *E. coli* strain harboring a studied plasmid. Then, 30 μL aliquots of the *E. coli* and cyanobacterial mixture were spotted onto non-selective MM agar plates that were incubated at 30°C for 72 h under light (1500 lux, 18.75 μE.m^–2^.s^–1^). Then, 4 spots were resuspended into 200 μL of fresh MM medium. One 50 μL-aliquot was serially diluted in and plated on fresh MM to calculate the number of *Cyanothece* PCC 7425 cells (colony-forming units), while the other three 50 μL-aliquots were plated onto MM containing the selective antibiotics and incubated for 10 days under standard conditions to select the conjugant clones, and calculate the frequency of trans-conjugation. A negative control was realized by spreading onto antibiotic-containing MM plates *Cyanothece* PCC 7425 cells that were not co-incubated with *E. coli* cells to detect possible spontaneous antibiotic resistant mutant cells. Antibiotics resistant conjugant clones were re-streaked onto selective plates, prior to analyzing their plasmid content through PCR and DNA sequencing (Eurofins Genomics, see Supplementary Table S2 for sequencing primers).

### Spectrophotometric Assay of Chloramphenicol Acetyl-Transferase Activity

CAT assays were done basically as described for *Synechocystis* PCC 6803 (Ferino and Chauvat, 1989; Marraccini et al., 1993). *Cyanothece* PCC 7425 strains harboring the pSB2A or pSB2T plasmids were grown in MM_CaC_ at 30°C under 2000 lux (1 × 10^8^ cells.mL^–1^) washed twice and resuspended into 2 mL of 50 mM Tris-HCl, pH 8.0. Approximately 2.10^9^ cells were broken in a chilled Eaton press (250 Mpa). Cell extracts were centrifuged (14,000 rpm, 4°C, 10 min) to remove cell debris and were either stored at −20°C until assay or used directly. 1–40 μL of cells extracts mixed with 200 μL of reaction solution (Tris HCl 100 mM pH 8, DTNB 0.4 mg.mL^–1^ and acetyl CoA 0.1 mM) were loaded into a 96-well plate (Greiner bio-one). Five μL of chloramphenicol at 5 mM was automatically added in each well by a microplate reader (ClarioStar; BMG Labtech). Immediately the absorption at 412 nm of the yellow TNB (5′-thio-2-nitrobenzoic acid) product was measured for 3 min at 30°C. Chloramphenicol acyltransferase activity was defined as the number of nanomoles of chloramphenicol acetylated per minute per mg protein that were quantified by the Bradford assay (Bio-Rad) using bovine serum albumin (0–10 μg BSA) as the standard.

### Spectrophotometric Assay of Beta-Galactosidase Activity

β-galactosidase assays were done basically as described for *Synechocystis* PCC 6803 (Mermet-Bouvier and Chauvat, 1994). *Cyanothece* PCC 7425 strains harboring the pFCI or pPMB13 plasmids grown at 30°C in MM_CaC_ under 2000 lux (5 × 10^7^ cells.mL^–1^) were heat-induced by a fourfold dilution into prewarmed growth medium and then incubated for 168 h at the indicated temperatures (30–39°C). Roughly, 2 × 10^9^ cells were washed, resuspended (in 2 mL Tris-HCl 50 mM pH 8.0), broken and centrifuged as described above. Immediately, 2–10 μL of cells extracts mixed with 200 μL of reaction solution (100 mM Na_2_HPO_4_/NaH_2_PO_4_ buffer pH 7.5, 50 mM β-mercaptoethanol) were loaded into a clear 96-well plate (Greiner bio-one). Then, 40 μL of ONPG (of *o*-nitrophenol-galactoside) 4 mg.mL^–1^ in K_2_HPO_4_/KH_2_PO_4_ buffer, pH 7.5) were automatically added in each well with a microplate reader (ClarioStar; BMG Labtech). The reaction was immediately followed by measuring the absorption at 420 nm of the yellow ONP (*o*-nitrophenol) product for 3 min at 30°C. β-galactosidase activity was calculated as the number of micromoles ONPG hydrolyzed per minute per mg proteins, which were quantified by the Bradford assay.

### Microscopy

Mid-log phase cultures of *Synechocystis* PCC 6803 and *Cyanothece* PCC 7425 cells harboring the pSB2TΔKm^R^-gfp, pSB2T-ccmk1_tsbp__1_-gfp and pSB2T-maf_S__6803_-gfp plasmids were placed in sandwiches consisting of two glass coverslips (22 mm diameter, Paul Marienfeld GmbH & Co. KG) one of which being coated with a Poly-L-lysine (Sigma-Aldrich) monolayer. These coverslip sandwiches were sealed and placed inside a home-made sample holder, which was mounted on a Nikon Ti-U inverted microscope coupled with an iXon ULTRA 897 CCD camera (Andor Technology), equipped with a 100x oil immersion (NA 1.45) microscope objective. Epifluorescence images were recorded using an excitation provided by a plasma light source (HPLS245 Thorlabs, Inc.) and an excitation filter (MF469-35 Thorlabs, Inc.), while for super-resolution laser scanning images a 488 nm laser (OBIS, Coherent) was used as an excitation source.

Chlorophyll and GFP fluorescence were recorded using ET655LP (Chroma Technology Corporation) and MF525/39 (Thorlabs, Inc.) emission filters, respectively.

### Limonene Collection and Quantification/Measurement by Gas Chromatography–Mass Spectrometry

*Cyanothece* PCC 7425 engineered for limonene production was grown photoautotrophically (1500–3500 lux) in 125- or 250-mL erlenmeyers respectively containing 25 or 50 mL cell suspensions overlaid with 20% (vol/vol) dodecane (analytical grade, Sigma-Aldrich) to trap limonene during the whole experimental time course. At time intervals, aliquots of 300 μL were harvested from the dodecane overlay. One μL samples were injected into a GC-MS [Trace1300 (GC) + ISQ LT (MS), ThermoScientific] equipped with a TG-5MS column (30 m × 0.25 mm × 0.25 μm) and operated with H_2_ carrier gas at 1.0 mL.min^–1^; ionization voltage 70 eV, split ratio of 25:1; transfer line temperature 250°C; ion source temperature 200°C. The oven program was as followed: 50°C (held for 1 min), 50–150°C (10°C min^–1^), 150–250°C (20°C min^–1^), and held for 5 min, with a total program of 21 min. Analysis were carried out in the selected ion monitoring mode: m/z = 50–650.

The limonene peak was identified based on its different ion chromatogram and retention time as compared to those of the α-pinene (Supelco 80599, Sigma-Aldrich) used as an internal standard. A standard curve at m/z = 136 was constructed by GC peak integration of serial dilutions of *S*-limonene (Supelco 62128, Sigma-Aldrich) pure standard in dodecane (Supplementary Figure S9). The limonene concentration in every dodecane sample was determined using this ratio and by taking-into-account the decrease of the dodecane overlay volume after each sample collection.

## Data Availability Statement

All datasets generated for this study are included in the article/Supplementary Material.

## Author Contributions

FC, CC-C, J-FS, and KB conceived the project. FC, CC-C, CC, ED-S, AG, BR, J-FS, and KB conceived the experiments. CC, ED-S, XK, SG, CI, SS, AG, EM, and D-AB performed the experiments. All authors analyzed the data, read and approved the manuscript. FC, CC-C, and CC drafted the manuscript. FC and CC-C wrote the manuscript. FC agreed to serve as the author responsible for contact and ensures communication.

## Conflict of Interest

The authors declare that the research was conducted in the absence of any commercial or financial relationships that could be construed as a potential conflict of interest.

## References

[B1] Bandyopadhyay A. Elvitigala T. Welsh E. Stöckel J. Liberton M. Min H. (2011). Novel metabolic attributes of the genus *Cyanothece*, comprising a group of unicellular nitrogen-fixing cyanobacteria. *mBio* 2:e00214-11. 10.1128/mBio.00214-11 21972240 PMC3187577

[B2] Blondeau M. Sachse M. Boulogne C. Gillet C. Guigner J. M. Skouri-Panet F. (2018). Amorphous calcium carbonate granules form within an intracellular compartment in calcifying cyanobacteria. *Front. Microbiol.* 9:1768. 10.3389/fmicb.2018.01768 30127775 PMC6087745

[B3] Cameron J. C. Wilson S. C. Bernstein S. L. Kerfeld C. A. (2013). Biogenesis of a bacterial organelle: the carboxysome assembly pathway. *Cell* 155 1131–1140. 10.1016/j.cell.2013.10.044 24267892

[B4] Casadaban M. J. Cohen S. N. (1980). Analysis of gene control signals by DNA fusion and cloning in *Escherichia coli*. *J. Mol. Biol.* 138 179–207. 10.1016/0022-2836(80)90283-1 6997493

[B5] Cassier-Chauvat C. Chauvat F. (2014). “Cell division in cyanobacteria,” in *The Cell Biology of Cyanobacteria*, eds Flores E. Herrero A. (Norfolk: Caister Academic Press).

[B6] Cassier-Chauvat C. Chauvat F. (2018). *Cyanobacteria: Wonderful Microorganisms for Basic and Applied Research.* Hoboken, NJ: John Wiley & Sons Ltd.

[B7] Cassier-Chauvat C. Dive V. Chauvat F. (2017). Cyanobacteria: photosynthetic factories combining biodiversity, radiation resistance, and genetics to facilitate drug discovery. *Appl. Microbiol. Biotechnol.* 101 1359–1364. 10.1007/s00253-017-8105-z 28083651

[B8] Cassier-Chauvat C. Veaudor T. Chauvat F. (2016). Comparative genomics of DNA recombination and repair in cyanobacteria: biotechnological implications. *Front. Microbiol.* 7:1809. 10.3389/fmicb.2016.01809 27881980 PMC5101192

[B9] Chauvat F. Corre B. Herdman M. Joset-Espardellier F. (1982). Energetic and metabolic requirements for the germination of akinetes of the cyanobacterium Nostoc PCC 7524. *Arch. Microbiol.* 133 44–49. 10.1007/BF00943768

[B10] Chuck C. J. Donnelly J. (2014). The compatibility of potential bioderived fuels with Jet A-1 aviation kerosene. *Appl. Energy* 118 83–91. 10.1016/j.apenergy.2013.12.019

[B11] Colby S. M. Alonso W. R. Katahira E. J. McGarvey D. J. Croteau R. (1993). 4S-limonene synthase from the oil glands of spearmint (*Mentha spicata*). cDNA isolation, characterization, and bacterial expression of the catalytically active monoterpene cyclase. *J. Biol. Chem.* 263 23016–23024. 8226816

[B12] Dai W. Chen M. Myers C. Ludtke S. J. Pettitt B. M. King J. A. (2018). Visualizing individual RuBisCO and its assembly into carboxysomes in marine cyanobacteria by cryo-electron tomography. *J. Mol. Biol.* 430 4156–4167. 10.1016/j.jmb.2018.08.013 30138616 PMC6252266

[B13] Davies F. K. Work V. H. Beliaev A. S. Posewitz M. C. (2014). Engineering limonene and bisabolene production in wild type and a glycogen-deficient mutant of *Synechococcus* sp. PCC 7002. *Front. Bioeng. Biotechnol.* 2:21. 10.3389/fbioe.2014.00021 25152894 PMC4126464

[B14] De Wever A. Benzerara K. Coutaud M. Caumes G. Poinsot M. Skouri-Panet F. (2019). Evidence of high Ca uptake by cyanobacteria forming intracellular CaCO3 and impact on their growth. *Geobiology* 17 676–690. 10.1111/gbi.12358 31347755

[B15] Demay J. Bernard C. Reinhardt A. Marie B. (2019). Natural products from cyanobacteria: focus on beneficial activities. *Mar. Drugs* 17:320. 10.3390/md17060320 31151260 PMC6627551

[B16] Domain F. Houot L. Chauvat F. Cassier-Chauvat C. (2004). Function and regulation of the cyanobacterial genes lexA, recA and ruvB: LexA is critical to the survival of cells facing inorganic carbon starvation. *Mol. Microbiol.* 53 65–80. 10.1111/j.1365-2958.2004.04100.x 15225304

[B17] Ducat D. C. Way J. C. Silver P. A. (2011). Engineering cyanobacteria to generate high-value products. *Trends Biotechnol.* 29 95–103. 10.1016/j.tibtech.2010.12.003 21211860

[B18] Dutheil J. Saenkham P. Sakr S. Leplat C. Ortega-Ramos M. Bottin H. (2012). The AbrB2 autorepressor, expressed from an atypical promoter, represses the hydrogenase operon to regulate hydrogen production in *Synechocystis* strain PCC6803. *J. Bacteriol.* 194 5423–5433. 10.1128/JB.00543-12 22865847 PMC3457224

[B19] Ferino F. Chauvat F. (1989). A promoter-probe vector-host system for the cyanobacterium, *Synechocystis* PCC6803. *Gene* 84 257–266. 10.1016/0378-1119(89)90499-X 2515116

[B20] Hamilton T. L. Bryant D. A. Macalady J. L. (2016). The role of biology in planetary evolution: cyanobacterial primary production in low-oxygen Proterozoic oceans. *Environ. Microbiol.* 18 325–340. 10.1111/1462-2920.13118 26549614 PMC5019231

[B21] Hamoen L. W. (2011). Cell division blockage: but this time by a surprisingly conserved protein. *Mol. Microbiol.* 81 1–3. 10.1111/j.1365-2958.2011.07693.x 21595759

[B22] Herrero A. Stavans J. Flores E. (2016). The multicellular nature of filamentous heterocyst-forming cyanobacteria. *FEMS Microbiol. Rev.* 40 831–854. 10.1093/femsre/fuw029 28204529

[B23] Houssen W. E. Koehnke J. Zollman D. Vendome J. Raab A. Smith M. C. M. (2012). The discovery of new cyanobactins from *Cyanothece* PCC 7425 defines a new signature for processing of patellamides. *ChemBioChem* 13 2683–2689. 10.1002/cbic.201200661 23169461

[B24] Jansson C. Northen T. (2010). Calcifying cyanobacteria-the potential of biomineralization for carbon capture and storage. *Curr. Opin. Biotechnol.* 21 365–371. 10.1016/j.copbio.2010.03.017 20456936

[B25] Jongedijk E. Cankar K. Buchhaupt M. Schrader J. Bouwmeester H. Beekwilder J. (2016). Biotechnological production of limonene in microorganisms. *Appl. Microbiol. Biotechnol.* 100 2927–2938. 10.1007/s00253-016-7337-7 26915992 PMC4786606

[B26] Klemke F. Nürnberg D. J. Ziegler K. Beyer G. Kahmann U. Lockau W. (2016). CphA2 is a novel type of cyanophycin synthetase in N2-fixing cyanobacteria. *Microbiology* 162 526–536. 10.1099/mic.0.000241 26781249

[B27] Knoot C. J. Ungerer J. Wangikar P. P. Pakrasi H. B. (2018). Cyanobacteria: Promising biocatalysts for sustainable chemical production. *J. Biol. Chem.* 293 5044–5052. 10.1074/jbc.R117.815886 28972147 PMC5892586

[B28] Koksharova O. A. Wolk C. P. (2002). A novel gene that bears a DnaJ motif influences cyanobacterial cell division. *J. Bacteriol.* 184 5524–5528. 10.1128/JB.184.19.5524-5528.2002 12218043 PMC135336

[B29] Legrand B. Miras Y. Beauger A. Dussauze M. Latour D. (2019). Akinetes and ancient DNA reveal toxic cyanobacterial recurrences and their potential for resurrection in a 6700-year-old core from a eutrophic lake. *Sci. Total Environ.* 687 1369–1380. 10.1016/j.scitotenv.2019.07.100 31412470

[B30] Liberton M. Bandyopadhyay A. Pakrasi H. B. (2019). Enhanced nitrogen fixation in a glgX-deficient strain of *Cyanothece* sp. strain ATCC 51142, a unicellular nitrogen-fixing cyanobacterium. *Appl. Environ. Microbiol.* 85:e02887-18. 10.1128/AEM.02887-18 30709817 PMC6585492

[B31] Lin P. C. Pakrasi H. B. (2019). Engineering cyanobacteria for production of terpenoids. *Planta* 249 145–154. 10.1007/s00425-018-3047-y 30465115

[B32] Lin P.-C. Saha R. Zhang F. Pakrasi H. B. (2017). Metabolic engineering of the pentose phosphate pathway for enhanced limonene production in the cyanobacterium *Synechocystis* sp. PCC 6803. *Sci. Rep.* 7:17503. 10.1038/s41598-017-17831-y 29235513 PMC5727528

[B33] MacCready J. S. Basalla J. L. Vecchiarelli A. G. (2020). Origin and evolution of carboxysome positioning systems in cyanobacteria. *Mol. Biol. Evol.* 37 1434–1451. 10.1093/molbev/msz308 31899489 PMC7182216

[B34] Marbouty M. Saguez C. Cassier-Chauvat C. Chauvat F. (2009). Characterization of the FtsZ-interacting septal proteins SepF and Ftn6 in the spherical-celled cyanobacterium *Synechocystis* strain PCC 6803. *J. Bacteriol.* 191 6178–6185. 10.1128/JB.00723-09 19648234 PMC2747883

[B35] Marraccini P. Bulteau S. Cassier-Chauvat C. Mermet-Bouvier P. Chauvat F. (1993). A conjugative plasmid vector for promoter analysis in several cyanobacteria of the genera *Synechococcus* and *Synechocystis*. *Plant Mol. Biol.* 23 905–909. 10.1007/BF00021546 8251644

[B36] Marteyn B. Domain F. Legrain P. Chauvat F. Cassier-Chauvat C. (2009). The thioredoxin reductase-glutaredoxins-ferredoxin crossroad pathway for selenate tolerance in *Synechocystis* PCC6803. *Mol. Microbiol.* 71 520–532. 10.1111/j.1365-2958.2008.06550.x 19040637

[B37] Mazouni K. Domain F. Cassier-Chauvat C. Chauvat F. (2004). Molecular analysis of the key cytokinetic components of cyanobacteria: FtsZ, ZipN and MinCDE. *Mol. Microbiol.* 52 1145–1158. 10.1111/j.1365-2958.2004.04042.x 15130131

[B38] Mermet-Bouvier P. Cassier-Chauvat C. Marraccini P. Chauvat F. (1993). Transfer and replication of RSF1010-derived plasmids in several cyanobacteria of the genera *Synechocystis* and *Synechococcus*. *Curr. Microbiol.* 27 323–327. 10.1007/BF01568955

[B39] Mermet-Bouvier P. Chauvat F. (1994). A conditional expression vector for the cyanobacteria *Synechocystis* sp. strains PCC6803 and PCC6714 or *Synechococcus* sp. strains PCC7942 and PCC6301. *Curr. Microbiol.* 28 145–148. 10.1007/BF01571055 7764699

[B40] Min H. Sherman L. A. (2010). Genetic transformation and mutagenesis via single-stranded DNA in the unicellular, diazotrophic cyanobacteria of the genus *Cyanothece*. *Appl. Environ. Microbiol.* 16 7641–7645. 10.1128/AEM.01456-10 20851971 PMC2976206

[B41] Montgomery B. L. (2015). Light-dependent governance of cell shape dimensions in cyanobacteria. *Front. Microbiol.* 6:514. 10.3389/fmicb.2015.00514 26074902 PMC4443024

[B42] Narainsamy K. Marteyn B. Sakr S. Cassier-Chauvat C. Chauvat F. (2013). “Genomics of the Pleïotropic Glutathione System in Cyanobacteria,” in *Advances in Botanical Research*, ed. Callow J. A. (Amsterdam: Elsevier), 157–188. 10.1016/b978-0-12-394313-2.00005-6

[B43] Ortega-Ramos M. Jittawuttipoka T. Saenkham P. Czarnecka-Kwasiborski A. Bottin H. Cassier-Chauvat C. (2014). Engineering *Synechocystis* PCC6803 for hydrogen production: influence on the tolerance to oxidative and sugar stresses. *PLoS One* 9:e0089372. 10.1371/journal.pone.0089372 24586727 PMC3933540

[B44] Ponce-Toledo R. I. Deschamps P. López-García P. Zivanovic Y. Benzerara K. Moreira D. (2017). An early-branching freshwater cyanobacterium at the origin of plastids. *Curr. Biol.* 27 386–391. 10.1016/j.cub.2016.11.056 28132810 PMC5650054

[B45] Porta D. Rippka R. Hernández-Mariné M. (2000). Unusual ultrastructural features in three strains of *Cyanothece* (cyanobacteria). *Arch. Microbiol.* 173 154–163. 10.1007/s002039900126 10795687

[B46] Rippka R. Deruelles J. Waterbury J. B. Herdman M. Stanier R. Y. (1979). Generic assignments, strain histories and properties of pure cultures of cyano- bacteria. *J. Gen. Microbiol.* 11 1–61. 10.1099/00221287-111-1-1

[B47] Sakamoto T. Delgaizo V. B. Bryant D. A. (1998). Growth on urea can trigger death and peroxidation of the cyanobacterium *Synechococcus* sp. strain PCC 7002. *Appl. Environ. Microbiol.* 64 2361–2366. 10.1128/AEM.64.7.2361-2366.1998 9647800 PMC106396

[B48] Sakr S. Dutheil J. Saenkham P. Bottin H. Leplat C. Ortega-Ramos M. (2013). The activity of the *Synechocystis* PCC6803 AbrB2 regulator of hydrogen production can be post-translationally controlled through glutathionylation. *Int. J. Hydrogen Energy* 38 13547–13555. 10.1016/j.ijhydene.2013.07.124

[B49] Schirrmeister B. E. Gugger M. Donoghue P. C. J. (2015). Cyanobacteria and the great oxidation event: evidence from genes and fossils. *Palaeontology* 58 769–785. 10.1111/pala.12178 26924853 PMC4755140

[B50] Singh J. S. Kumar A. Rai A. N. Singh D. P. (2016). Cyanobacteria: a precious bio-resource in agriculture, ecosystem, and environmental sustainability. *Front. Microbiol.* 7:529. 10.3389/fmicb.2016.00529 27148218 PMC4838734

[B51] Sommer M. Sutter M. Gupta S. Kirst H. Turmo A. Lechno-Yossef S. (2019). Heterohexamers formed by CcmK3 and CcmK4 increase the complexity of beta carboxysome shells. *Plant Physiol.* 179 156–167. 10.1104/pp.18.01190 30389783 PMC6324227

[B52] Stanier R. Y. Kunisawa R. Mandel M. Cohen-Bazire G. (1971). Purification and properties of unicellular blue-green algae (order Chroococcales). *Bacteriol. Rev.* 35 171–205. 10.1128/mmbr.35.2.171-205.1971 4998365 PMC378380

[B53] Sun Y. Wollman A. J. M. Huang F. Leake M. C. Liu L. N. (2019). Single-organelle quantification reveals stoichiometric and structural variability of carboxysomes dependent on the environment. *Plant Cell* 31 1648–1664. 10.1105/tpc.18.00787 31048338 PMC6635877

[B54] Tracy N. I. Chen D. Crunkleton D. W. Price G. L. (2009). Hydrogenated monoterpenes as diesel fuel additives. *Fuel* 88 2238–2240. 10.1016/j.fuel.2009.02.002

[B55] Veaudor T. Cassier-Chauvat C. Chauvat F. (2018). Overproduction of the cyanobacterial hydrogenase and selection of a mutant thriving on urea, as a possible step towards the future production of hydrogen coupled with water treatment. *PLoS One* 13:e0198836. 10.1371/journal.pone.0198836 29879209 PMC5991728

[B56] Veaudor T. Cassier-Chauvat C. Chauvat F. (2019). Genomics of urea transport and catabolism in cyanobacteria: biotechnological implications. *Front. Microbiol.* 10:2052. 10.3389/fmicb.2019.02052 31551986 PMC6737895

[B57] Wang X. Liu W. Xin C. Zheng Y. Cheng Y. Sun S. (2016). Enhanced limonene production in cyanobacteria reveals photosynthesis limitations. *Proc. Natl. Acad. Sci. U.S.A.* 113 14225–14230. 10.1073/pnas.1613340113 27911807 PMC5167140

