# Supplementary material for: A Genetic Toolbox for the New Model Cyanobacterium Cyanothece PCC 7425: A Case Study for the Photosynthetic Production of Limonene
Source: Front Microbiol. 2020 Sep 18;11:586601. doi: 10.3389/fmicb.2020.586601 (PMC7530172; doi:10.3389/fmicb.2020.586601)

Supplementary Figure S1. Influence of nitrogen sources on the growth of *Cyanothece* PCC 7425.

Cells inoculated at $OD_{750} = 0.1$ were grown at 30°C, under light (2000 lux), in liquid nitrogen-free mineral medium (-N), which was in some cases supplemented with 2 to 17 mM nitrate ($NaNO_3$; 17 mM is the concentration of the standard medium) or with 1 to 4 mM urea ($CO(NH_2)_2$), as indicated. Urea and nitrate were added either all at once or by little additions of small doses (1 mM Urea or 2 mM $NaNO_3$) of equal nitrogen content (2 mM N) at days 0, 3, 7 and 10 (as indicated in red). Cells growing on nitrate, the usual nitrogen source for most cyanobacteria, cared only about nitrate concentration (the greater, the better) whereas cells growing on urea required successive additions of small doses, rather than a brutal addition of a high dose which could be toxic.

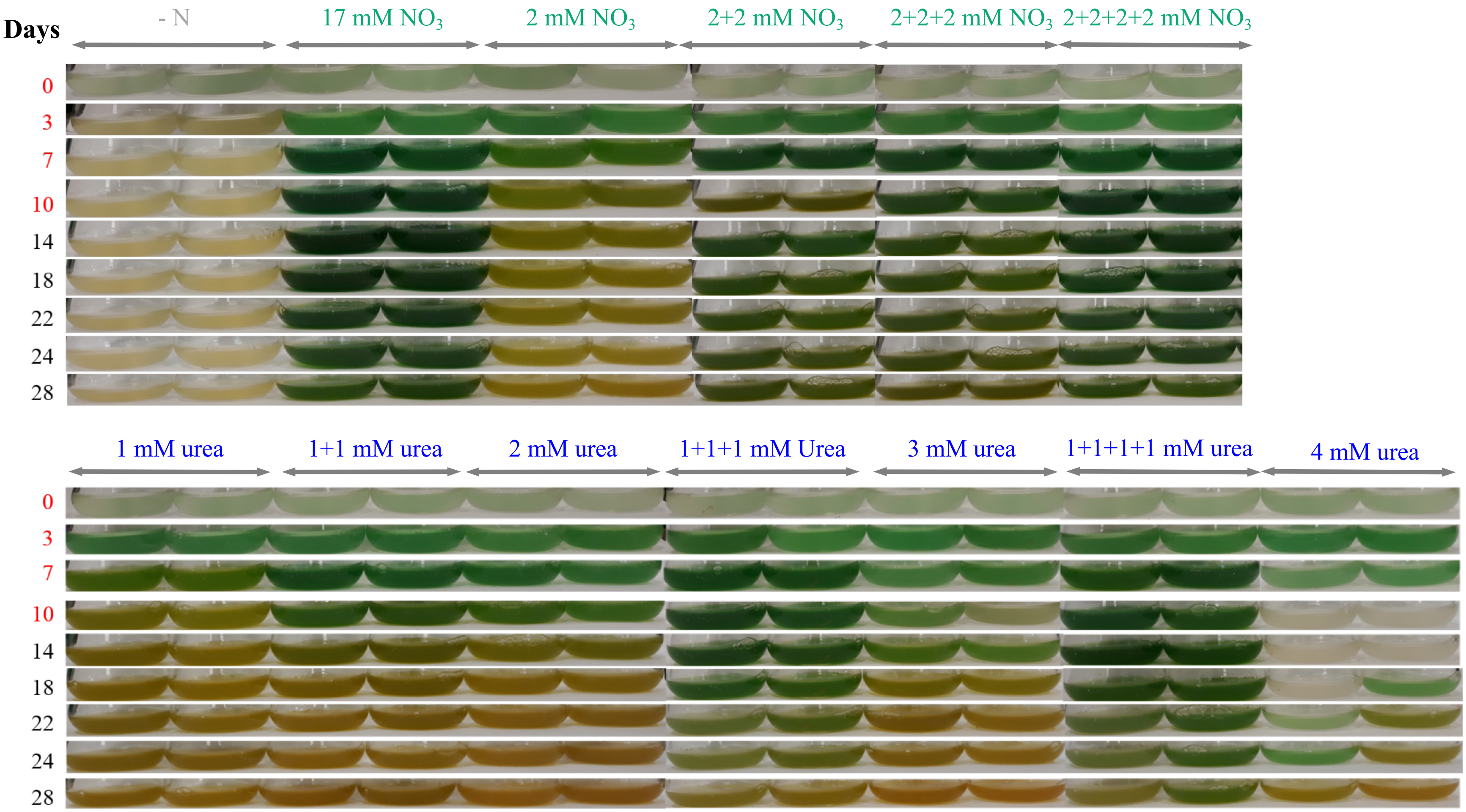

Supplement: Supplementary file 1 [file Presentation_1.pdf]
