# Supplementary material for: A Genetic Toolbox for the New Model Cyanobacterium Cyanothece PCC 7425: A Case Study for the Photosynthetic Production of Limonene
Source: Front Microbiol. 2020 Sep 18;11:586601. doi: 10.3389/fmicb.2020.586601 (PMC7530172; doi:10.3389/fmicb.2020.586601)

Supplementary Figure S2. Schematic representation and PCR analysis of the pSB2A-plasmid family replicating in *Cyanothece* PCC 7425

(A) Schematic representation of the pSB2A plasmid and its derivatives. The genes are represented by colored arrows pointing into the direction of their transcription. The PCR primers are colored in red and their corresponding PCR are indicated by double arrows. (B) Typical UV-light image of the agarose gel showing the relevant PCR products. M (marker DNA) indicates the GeneRuler 1 kb Plus DNA Ladder (Thermo Scientific).

A

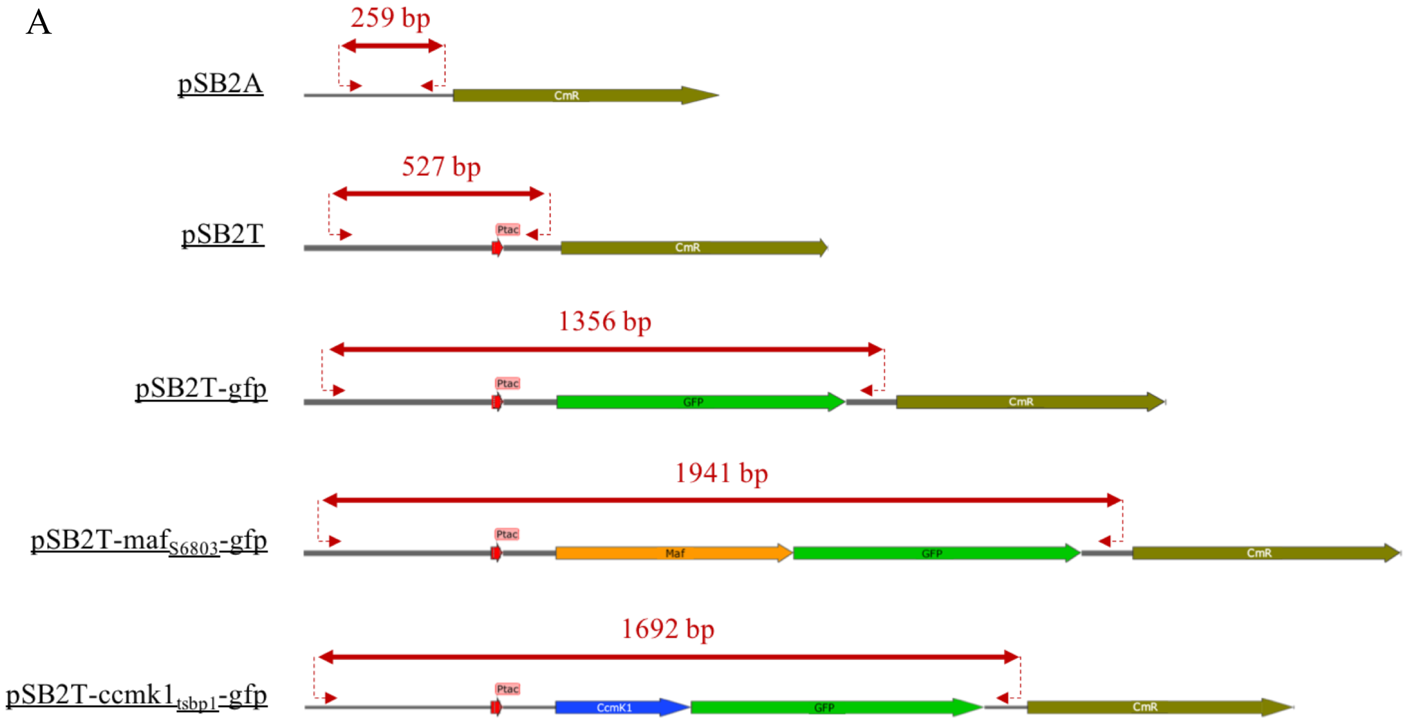

B

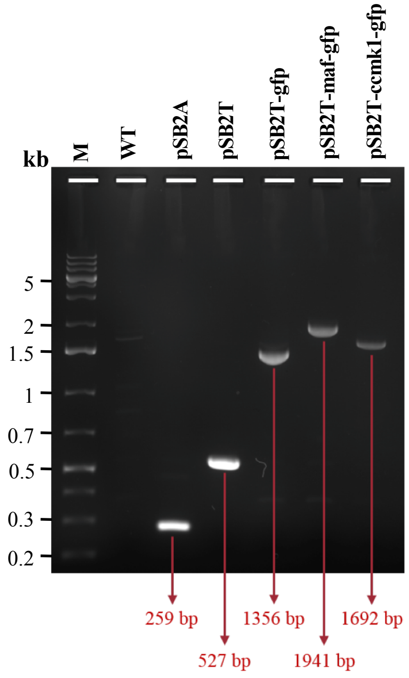

Supplement: Supplementary file 2 [file Presentation_2.pdf]
