# Supplementary material for: A Genetic Toolbox for the New Model Cyanobacterium Cyanothece PCC 7425: A Case Study for the Photosynthetic Production of Limonene
Source: Front Microbiol. 2020 Sep 18;11:586601. doi: 10.3389/fmicb.2020.586601 (PMC7530172; doi:10.3389/fmicb.2020.586601)
Supplement: Supplementary file 5 [file Presentation_5.pdf]

**Supplementary Figure S5 -Relevant part of the nucleotide sequence of the plasmid pSB2T-maf$_{S6803}$-gfp.** The DNA segment encompassing the *tac* promoter is written in red cases showing the so-called -35 (5'-TTGACA-3') and -10 (5'-TATAAT-3') promoter boxes, and the A transcription start site in upper cases. The maf$_{S6803}$ and **gfp** part of the maf-fusion gene are colored in blue and green. GTTATC and GATAAC sequences are the scars corresponding to the cloning of the *Eco*RV DNA fragment harboring the maf$_{S6803}$-gfp fusion gene into the pSB2T plasmid opened at its unique *Hpa*I site.The cleavage site for *Hpa*I and *Eco*RV are respectively: ***Hpa*I, GTT/AAC** and ***Eco*RV, GAT/ATC**.

gatccgggcttatcgactgcacggtgcaccaatgcttctggcgtcaggcagccatcggaagctgtggtatggctgtgcaggtcgtaaatcactgcataattc
gtgtcgctcaaggcgcactcccgttctggataatgttttttgcgccgacatcataacggttctggcaaatattctgaaatgagctg**TTGACA**attaatcatc
ggctcg**TATAAT**gtgtgga**A**ttgtgagcggataacaatttcacacaggaaacagaattcccggggatctccgcg**GTTATC**gttgtcccgatggaggcttcat
gaggcaatctctctactgtttttttgagaAGAGGAaacatctATGCCTCCAACTTTTGTTCTTGCTTCTGCTTCCCCCGCCCGCAAACGTTTGCTGGAAATG
GCCGGCATTAGCCCCCTAGTGGCCGTGAGCCATTTCGATGAATCTTCCTTAGCCGCCGACAACACAGTGGCCCTGGTGGAAGCTCTGGCCAAGGCTAAAGCA
GGAACGGTGGCCAGTAAATTCGCTGATGCCCTCGTCCTCGGCTGTGATTCCCTTTTATTGGTTAATGGTCAAACCTACGGTAAACCAGAGTCGCCCGCGGTG
GCGATCGCCCGTTGGCAAACCATGCGGGGTCAGGTGGGGGAACTGTACACGGGCCATGCCTTGATTGACCGCACCCAAAACCGTTGCCTGTGCCAAACGGGG
TTAACCAAAGTTCATTTTGCTGATGTGGATGACGACACCATCCAAGCCTACGTGGGTAGTGGGGAACCCCTCCAATGTGCTGGGGCGTTTGCCCTGGAAGGC
AAAGGGGGAATGTTAATTAATAAACTGGATGGTTGTTCCAGCAACGTGATTGGCCTGAGCTTGCCCATTCTGCGGTCATTGTTGCAACGGTTGGGTTACTCC
CTTAAAGATTTTTGGTCTAAAGGTGAAGAATTATTCACTGGTGTTGTCCCAATTTTGGTTGAATTAGATGGTGATGTTAATGGTCACAAATTTTCTGTCTCC
GGTGAAGGTGAAGGTGATGCTACTTACGGTAAATTGACCTTAAAATTTATTTGTACTACTGGTAAATTGCCAGTTCCATGGCCAACCTTAGTCACTACTTTC
GGTTATGGTGTTCAATGTTTTGCGAGATACCCAGATCATATGAAACAACATGACTTTTTCAAGTCTGCCATGCCAGAAGGTTATGTTCAAGAAAGAACTATT
TTTTTCAAAGATGACGGTAACTACAAGACCAGAGCTGAAGTCAAGTTTGAAGGTGATACCTTAGTTAATAGAATCGAATTAAAAGGTATTGATTTTAAAGAA
GATGGTAACATTTTAGGTCACAAATTGGAATACAACTATAACTCTCACAATGTTTACATCATGGCTGACAAACAAAAGAATGGTATCAAAGTTAACTTCAAA
ATTAGACACAACATTGAAGATGGTTCTGTTCAATTAGCTGACCATTATCAACAAAATACTCCAATTGGTGATGGTCCAGTCTTGTTACCAGACAACCATTAC
TTATCCACTCAATCTGCCTTATCCAAAGATCCAAACGAAAAGAGAGACCACATGGTCTTGTTAGAATTTGTTACTGCTGCTGGTATTACCCATGGTATGGAT
GAATTGTACAAACCCGGGGATGTAGGG**TAA**taatgctggccatgggctagtgagtttgatagtttcataattgtccacggcagggtttaagccctgcttttt
tgttgcccattggcaaactccaagacagccAAGCCGAATTCTGCA**GATAAC**

**Amino-acids sequence of the Maf$_{S6803}$-GFP fusion protein encoded by the pSB2T-maf$_{S6803}$-gfp plasmid**

MPPTFVLASASPARKRLLEMAGISPLVAVSHFDESSLAADNTVALVEALAKAKAGTVASKFADALVLGCDSLLLVNGQTYGKPESPAVAIARWQTMRGQVGE
LYTGHALIDRTQNRCLCQTGLTKVHFADVDDDTIQAYVGSGEPLQCAGAFALEGKGGMLINKLDGCSSNVIGLSLPILRSLLQRLGYSLKDFWSKGEELFTG
VVPILVELDGDVNGHKFSVSGEGEGDATYGKLTLKFICTTGKLPVPWPTLVTTFGYGVQCFARYPDHMKQHDFFKSAMPEGYVQERTIFFKDDGNYKTRAEV
KFEGDTLVNRIELKGIDFKEDGNILGHKLEYNYNSHNVYIMADKQKNGIKVNFKIRHNIEDGSVQLADHYQQNTPIGDGPVLLPDNHYLSTQSALSKDPNEK
RDHMVLLEFVTAAGITHGMDELYKPGDVG*
