# Supplementary material for: A Genetic Toolbox for the New Model Cyanobacterium Cyanothece PCC 7425: A Case Study for the Photosynthetic Production of Limonene
Source: Front Microbiol. 2020 Sep 18;11:586601. doi: 10.3389/fmicb.2020.586601 (PMC7530172; doi:10.3389/fmicb.2020.586601)

Supplementary Figure S6 -- Construction of the pC-LS plasmid for constitutive expression of the *Mentha spicata* 4S-limonene synthase encoding gene adapted to the *Synechocystis* PCC 6803 codon usage. The *Nde*I and *EcoR*I restriction sites are used for the cloning the *limonene synthase* gene from the pEX_K4_LS (provided by Eurofins Genomics) into the pC vector, generating the pC-LS plasmid. The *limonene synthase* gene (shown in yellow) is expressed constitutively from the strong λ phage *pR* promoter (shown in red). The genes are represented by colored arrows pointing into the direction of their transcription. Note that the Cm^R gene of pC-LS is truncated and that the Sp^R/Sm^R gene (pink) is flanked by the double terminator (TT, orange) to prevent readthrough of gene expression.

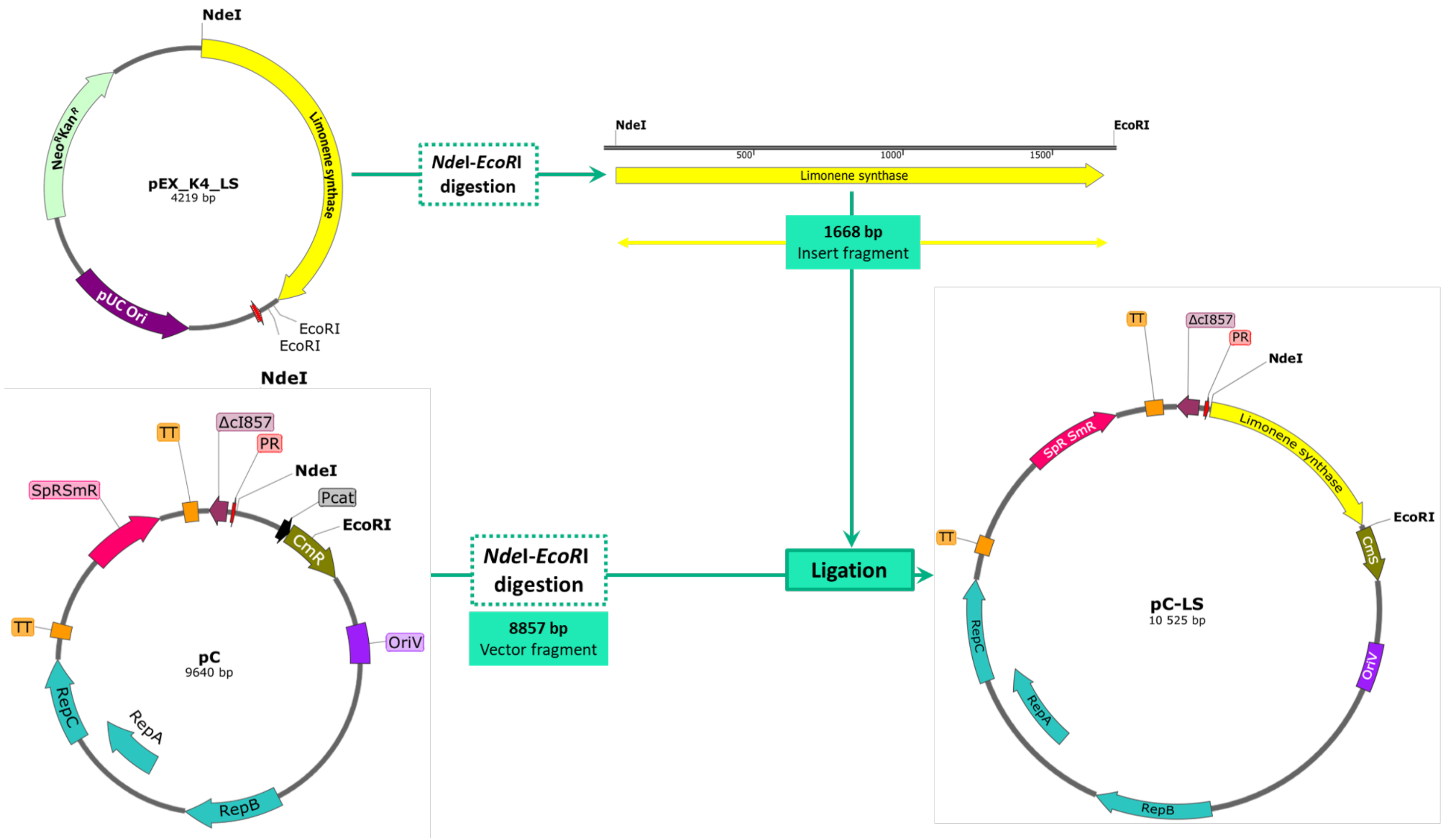

Supplement: Supplementary file 6 [file Presentation_6.pdf]
