# Supplementary material for: A Genetic Toolbox for the New Model Cyanobacterium Cyanothece PCC 7425: A Case Study for the Photosynthetic Production of Limonene
Source: Front Microbiol. 2020 Sep 18;11:586601. doi: 10.3389/fmicb.2020.586601 (PMC7530172; doi:10.3389/fmicb.2020.586601)
Supplement: Supplementary file 7 [file Presentation_7.pdf]

Supplementary Figure S7 -- Relevant part of the nucleotide sequence of the pC-LS plasmid expressing the limonene synthase gene from *Mentha spicata*. The lambda phage P_R promoter that comprises the -35 box (5'-**TTGACT-3'**), the -10 box (5'-**GATAAT-3'**) and the transcription start site (**A**) as well as the Shine d'Algarno sequence (ribosome bind site; **aaggagg**) sequence originating from the lambda phage *Cro* gene are shown in red (see (Mermet-Bouvier and Chauvat, 1994). The *Mentha spicata limonene synthase encoding gene* was adapted to *Synechocystis* PCC 6803 codon-usage and cloned in the *Nde*I (**catATG**) and *EcoR*I (**gaattc**) restriction thereby truncating the **chloramphenicol resistance gene** (Cm^S). This sequence can be amplified by the pFC1 Fw and pFC1 Rv primers.

5'-
GGCGACGTGCGTCCTCAAGCtgctcttgtgttaatggtttctttttgtgctcatacgttaaatctatcaccgcaagggataaatatctaacaccgtgcgtg**TTGACT**at
tttacctctggcggt**GATAAT**ggttgc**A**tgtact**aaggagg**t**catATG**GAGCGAAGAAGTGGCAACTACAACCCCTCGAGATGGGATGTTAATTTTATCCAGTCATTACT
ATCTGACTATAAAGAAGACAAACATGTGATCAGAGCGAGCGAACTAGTTACTTTAGTAAAGATGGAGCTGGAGAAAGAAACGGATCAGATCCGACAGCTAGAACTGATAG
ATGATCTTCAACGCATGGGCCTATCCGACCATTTTCAGAACGAATTTAAAGAAATATTGAGCTCAATTTACCTAGATCACCATTATTATAAGAACCCGTTTCCGAAAGAA
GAACGCGACCTTTACTCCACTTCATTAGCTTTTCGTCTCCTAAGGGAACACGGATTCCAAGTTGCGCAAGAGGTATTCGACAGTTTTAAAAACGAGGAAGGTGAGTTTAA
AGAGTCTTTATCTGACGACACTCGGGGATTACTTCAATTATATGAGGCCAGCTTTCTGCTAACGGAGGGTGAAACGACATTGGAGTCTGCAAGGGAGTTCGCCACTAAGT
TCTTAGAGGAGAAAGTAAATGAAGGAGGCGTTGACGGAGATCTGTTAACTCGAATTGCCTATAGCCTCGATATCCCGCTCCATTGGAGAATCAAACGTCCAAATGCCCCC
GTTTGGATTGAATGGTACCGTAAAAGGCCTGACATGAACCCAGTTGTGTTGGAACTTGCCATCCTGGACCTTAACATTGTGCAGGCCCAATTTCAAGAAGAACTGAAGGA
ATCCTTTCGCTGGTGGCGGAATACCGGCTTTGTTGAAAAACTCCCTTTTGCACGGGATCGGTTAGTGGAGTGCTACTTTTGGAACACCGGGATCATTGAACCTCGACAGC
ATGCCTCGGCTCGCATCATGATGGGCAAGGTTAATGCGCTCATTACTGTAATTGATGATATTTATGACGTCTACGGCACATTAGAAGAACTCGAACAATTTACCGATCTC
ATTCGCCGTTGGGATATTAATTCAATTGATCAGTTGCCGGACTACATGCAACTTTGCTTTTTAGCCTTAAATAATTTTGTGGACGACACAAGCTATGATGTGATGAAAGA
AAAAGGGGTCAATGTGATTCCATACTTGCGGCAATCTTGGGTTGACTTGGCCGATAAGTACATGGTCGAAGCACGTTGGTTTTATGGGGGCCATAAACCCTCCTTAGAAG
AATATTTGGAGAATAGCTGGCAAAGTATTTCCGGTCCCTGTATGCTGACCCACATTTTCTTCCGGGTCACCGATAGTTTCACCAAGGAAACCGTCGATAGTTTGTATAAG
TATCACGATCTGGTCCGCTGGTCCTCCTTCGTGCTGCGGTTGGCCGATGATTTGGGGACCAGTGTGGAAGAAGTGAGTCGTGGGGATGTGCCCAAATCCTTGCAATGTTA
TATGTCCGATTATAATGCCTCCGAAGCGGAAGCTCGGAAACATGTGAAGTGGCTAATTGCTGAAGTGTGGAAGAAAATGAATGCTGAACGGGTGAGTAAAGATAGTCCCT
TTGGCAAAGATTTTATTGGGTGTGCCGTGGATTTGGGTCGTATGGCGCAATTGATGTATCATAATGGTGATGGTCATGGTACCCAACACCCCATTATTCACCAACAAATG
ACCCGCACCTTGTTTGAGCCCTTTGCTTAAggatccatggtcgcgatcggtcgacgctagc**gaattc****CGTATGGCAATGAAAGACGGTGAGCTGGTGATATGGGATAGTG**
TTCACCCTTGTTACAC-3'

Amino acid sequence of the 4S-limonene synthase from *Mentha Spicata*

MERRSGNYNPSRWDVNFIQSLLSDYKEDKHVIRASELVTLVKMELEKETDQIRQLELIDDLQRMGLSDHFQNEFKEILSSIYLDHHYYKNPFPKEERDLYSTSLAFRLLREH
GFQVAQEVFDSFKNEEGEFKESLSDDTRGLLQLYEASFLLTEGETTLESAREFATKFLEEKVNEGGVDGDLLTRIAYSLDIPLHWRIKRPNAPVWIEWYRKRPDMNPVVLE
LAILDLNIVQAQFQEELKESFRWWRNTGFVEKLPFARDRLVECYFWNTGIIEPRQHASARIMMGKVNALITVIDDIYDVYGTLEELEQFTDLIRRWDINSIDQLPDYMQLCF
LALNNFVDDTSYDVMKEKGVNVIPYLRQSWVDLADKYMVEARWFYGGHKPSLEEYLENSWQSISGPCMLTHIFFRVTDSFTKETVDSLYKYHDLVRWSSFVLRLADDL
GTSVEEVSRGDVPKSLQCYMSDYNASEAEARKHVKWLIAEVWKKMNAERVSKDSPFGKDFIGCAVDLGRMAQLMYHNGDGHGTQHPIIHQQMTRTLFEPFA*
