# Supplementary material for: A Genetic Toolbox for the New Model Cyanobacterium Cyanothece PCC 7425: A Case Study for the Photosynthetic Production of Limonene
Source: Front Microbiol. 2020 Sep 18;11:586601. doi: 10.3389/fmicb.2020.586601 (PMC7530172; doi:10.3389/fmicb.2020.586601)
Supplement: Supplementary file 8 [file Presentation_8.pdf]

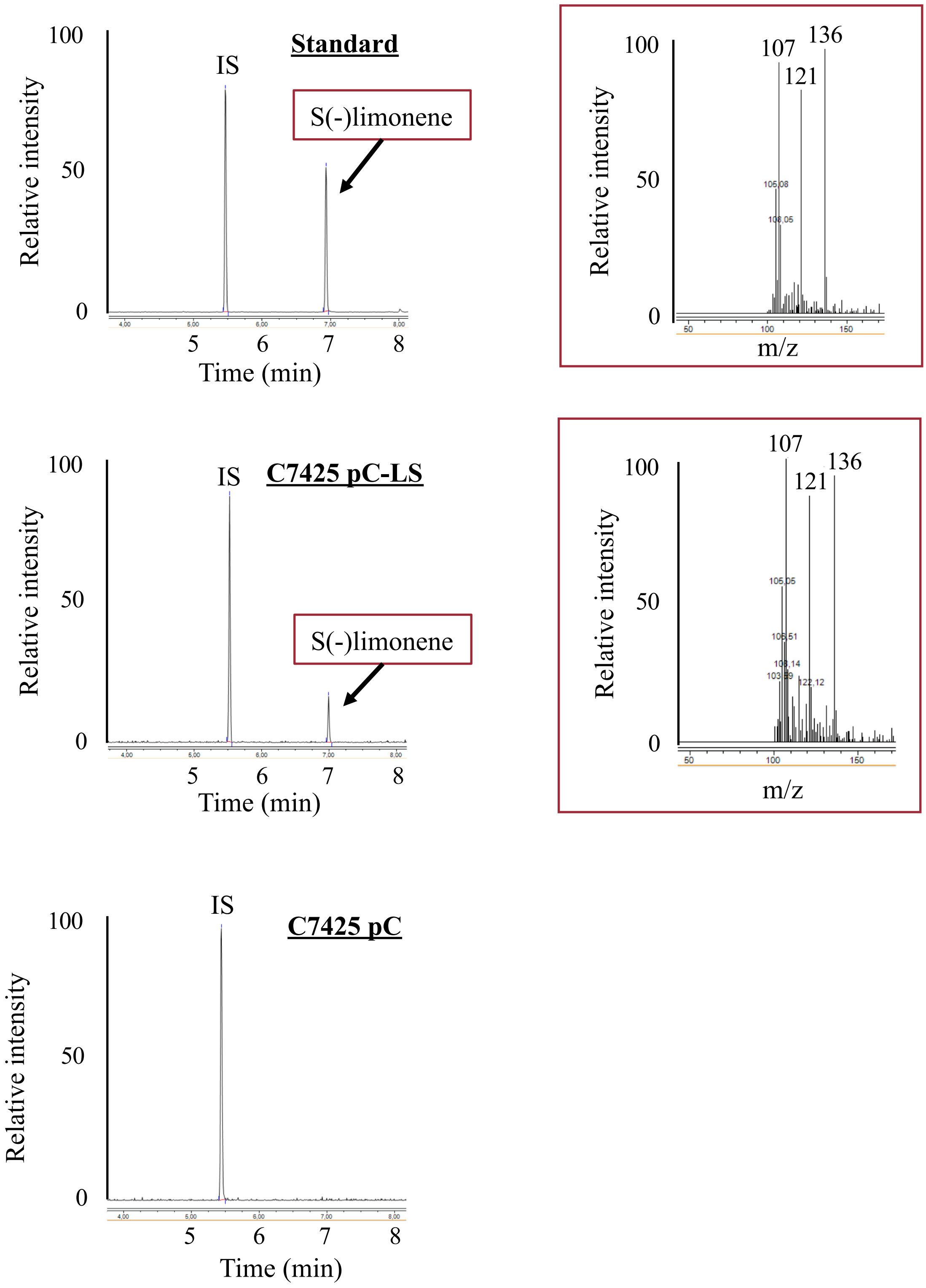

Supplementary Figure S8. GC–MS analyses of the dodecane overlays of the cultures of *Cyanothece* PCC 7425 strains harboring the pC or pC-LS plasmids. Ion chromatograms (left panels) and corresponding mass spectra (right panels) obtained from GC-MS analyses of an authentic S-(-)-limonene standard or dodecane samples from cultures of Cyanothece PCC 7425 harboring either the empty pC (used as a negative control) or the pC-LS plasmid for limonene production. α-pinene (RT = 5.44 min) at 0.01 g/L was used as the internal standard (IS) for quantification.
