# Supplementary material for: A Genetic Toolbox for the New Model Cyanobacterium Cyanothece PCC 7425: A Case Study for the Photosynthetic Production of Limonene
Source: Front Microbiol. 2020 Sep 18;11:586601. doi: 10.3389/fmicb.2020.586601 (PMC7530172; doi:10.3389/fmicb.2020.586601)

Supplementary Figure S9 -Standard curve used to calculate the concentration of limonene in the dodecane overlay of cultures of the engineered *Cyanothece* PCC 7425 strain. Limonene (increasing concentrations) and pinene (fixed concentration) were spiked in dodecane, prior to GC/MS analysis.

**Concentration of S-limonene (mg.L^{-1}) in dodecane**	**0.1**	**0.3**	**0.5**	**0.8**	**1.0**	**2.5**	**5.0**	**7.5**	**10.0**	**25.0**	**50.0**	**75.0**	**100.0**
Area S-limonene	519	1322	2142	3612	4826	9281	12943	30328	43585	90489	216217	266942	419115
	567	1176	2486	3479	4472	11960	22828	28412	49068	124945	173189	354735	297089
	592	1144	2232	3736	4090	12514	22758	34639	39393	88345	213968	259314	335784
Area alpha-pinene at 10 mg.L^{-1} (IS)	36668	36685	36469	36084	36191	27154	19379	32766	30867	25723	28454	24956	28869
	34471	35773	38129	36954	36972	35469	34204	28788	35473	35011	23670	32848	20797
	36976	36994	35830	36092	29555	37414	34237	34121	28192	25598	28543	24919	22240
Area S-limonene / Area alpha-pinene	0.014	0.036	0.059	0.100	0.133	0.342	0.668	0.926	1.412	3.518	7.599	10.697	14.518
	0.016	0.033	0.065	0.094	0.121	0.337	0.667	0.987	1.383	3.569	7.317	10.799	14.285
	0.016	0.031	0.062	0.104	0.138	0.334	0.665	1.015	1.397	3.451	7.496	10.406	15.098
**Average**	**0.016**	**0.033**	**0.062**	**0.099**	**0.131**	**0.338**	**0.667**	**0.976**	**1.398**	**3.513**	**7.471**	**10.634**	**14.634**
**Standard deviation**	**0.001**	**0.003**	**0.003**	**0.005**	**0.009**	**0.004**	**0.002**	**0.046**	**0.014**	**0.059**	**0.143**	**0.204**	**0.419**

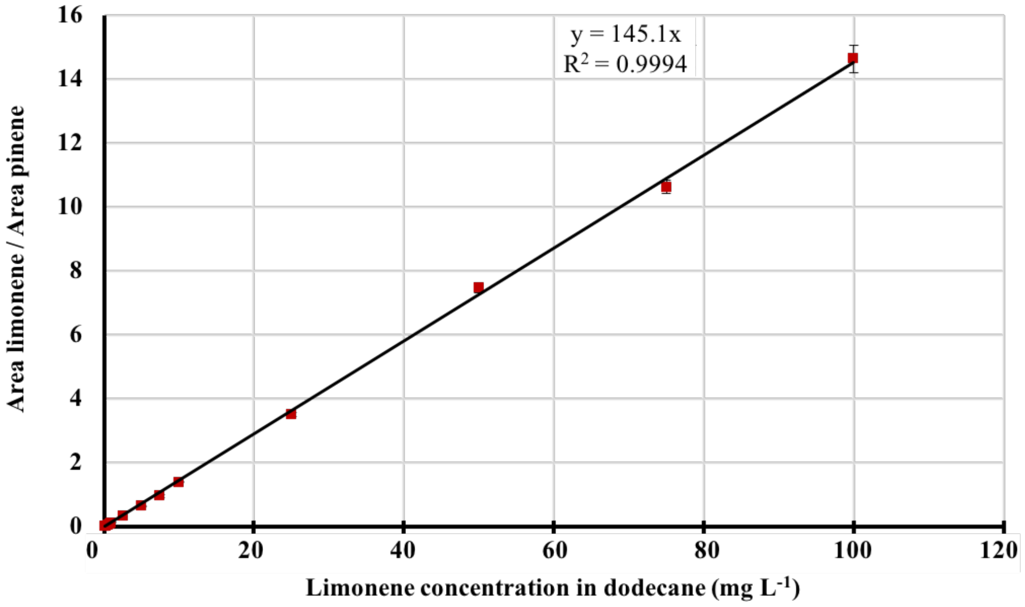

Supplement: Supplementary file 9 [file Presentation_9.pdf]
