# Supplementary material for: A Genetic Toolbox for the New Model Cyanobacterium Cyanothece PCC 7425: A Case Study for the Photosynthetic Production of Limonene
Source: Front Microbiol. 2020 Sep 18;11:586601. doi: 10.3389/fmicb.2020.586601 (PMC7530172; doi:10.3389/fmicb.2020.586601)
Supplement: Supplementary file 10 [file Table_1.docx]

| ***Supplementary Material***  **Supplementary Table S1 – Characteristics of the bacterial strains and plasmids used in this study** |
| --- |

| Strain/Plasmid | Relevant features | Reference |
| --- | --- | --- |
| *Escherichia coli CM404* | *E. coli* strain for conjugation. It harbors the self-transmissible pRK2013 plasmid | (Marraccini et al., 1993) |
| *Escherichia coli TOP10* | *E. coli* strain for cloning and conjugation | Invitrogen |
| *Escherichia coli MC1061* | *E. coli* strain for conjugation | (Casadaban and Cohen, 1980) |
| *Escherichia coli XL1 Blue* | *E. coli* strain for cloning and conjugation | Agilent |
| *Synechocystis* PCC 6803 | Best-studied cyanobacterium used as a genetic model | Pasteur Institute (Paris, France) |
| *Cyanothece* PCC 7425 | Unicellular cyanobacterium endowed with attractive properties | Pasteur Institute (Paris, France) |
| pFCI | RSF1010-derived plasmid vector (Sp^R^/Sm^R^,Cm^R^) harboring the strong *p*_R_ promoter tightly controlled by the temperature-sensitive repressor encoded by the *cI_857_* gene, for temperature-controlled protein production | (Mermet-Bouvier and Chauvat, 1994) |
| pPMB13 | pFCI derivative harboring the *E. coli* *lacZ* gene for temperature-controlled production of the beta-galactosidase reporter enzyme | (Mermet-Bouvier and Chauvat, 1994) |
| pC | pFCI derivative harboring a truncated *cI_857_* repressor gene for constitutive gene expression | (Veaudor et al., 2018) |
| pEX-K4-LS | Km^R^ plasmid harboring the Mentha spicata 4S-limonene synthase encoding gene adapted to the cyanobacterial codon usage flanked by *Nde*I and *EcoR*I restriction sites | This study and Eurofins Genomics |
| pC-LS | pC derivative plasmid (Sp/Sm^R^, Cm^S^) with the 4S-limonene synthase encoding gene cloned in between the *Nde*I and *EcoR*I sites | This study |
| pSB2A | RSF1010-derived plasmid vector (Sp^R^/Sm^R^, Km^R^,Cm^S^) harboring the promoter-less *cat* coding sequence (CS), which, when expressed, produces the chloramphenicol-acetyl-transferase reporter enzyme | (Marraccini et al., 1993) |
| pSB2T | pSB2A derivative (Sp^R^/Sm^R^, Km^R^, Cm^R^) expressing the *cat* CS from the strong the *E. coli* *tac* promoter, yielding a strong chloramphenicol-acetyl-transferase reporter activity | (Marraccini et al., 1993) |
| pSB2T-gfp | Km^S^ derivative of pSB2T strongly expressing the GFP coding sequence for strong production of the green-fluorescent reporter protein | (Mazouni et al., 2004) |
| pSB2T-maf_S6803_-gfp | pSB2T-derivative (Sp/Sm^R^, Km^R^) for strong production of the *Synechocystis* Maf protein translationally-fused to GFP | This study |
| pSB2AT-ccmk1_tsbp1_-gfp | pSB2T-derivative (Sp/Sm^R^, Km^R^) for strong production of the cyanobacterial CcmK1 carboxysome protein translationally-fused to GFP | This study and Genecust |
