# Supplementary material for: A Genetic Toolbox for the New Model Cyanobacterium Cyanothece PCC 7425: A Case Study for the Photosynthetic Production of Limonene
Source: Front Microbiol. 2020 Sep 18;11:586601. doi: 10.3389/fmicb.2020.586601 (PMC7530172; doi:10.3389/fmicb.2020.586601)
Supplement: Supplementary file 11 [file Table_2.docx]

**Supplementary Table S2 – List and characteristics of the PCR primers used in this study**

| Name | Sequence (5’-3’) | Purpose |
| --- | --- | --- |
| pFC1 and pFCI derivates | | |
| pFC1 Fw | GGCGACGTGCGTCCTCAAGC | PCR amplification and/or DNA sequencing of genes cloned in between the *Nde*I and *EcoR*I restriction sites of pFC1 and pC |
| pFC1 Rv | GTGTAACAAGGGTGAACAC |  |
| pSB2A and pSB2A derivates | | |
| pSB2A Fw | TAGCGAGGGCTTTACTAAGC | PCR amplification of the promoters or the genes cloned in pSB2A or its pSB2T derivative |
| pSB2A Rv | GCTCCTGAAAATCTCGTCG |  |
| Limonene synthase gene | | |
| LSF1 | GCGGAATACCGGCTTTGTTG | DNA sequencing of the 4S-limonene synthase encoding gene propagated in pC-LS |
| LSR1 | ACTATCGGTGACCCGGAAGA |  |
| LSR2 | TAACCGATCCCGTGCAAAAG |  |

Fw: forward; Rv: reverse
