# Supplementary material for: A Genetic Toolbox for the New Model Cyanobacterium Cyanothece PCC 7425: A Case Study for the Photosynthetic Production of Limonene
Source: Front Microbiol. 2020 Sep 18;11:586601. doi: 10.3389/fmicb.2020.586601 (PMC7530172; doi:10.3389/fmicb.2020.586601)
Supplement: Supplementary file 4 [file Presentation_4.pdf]

frontiers

Supplementary Material

Supplementary Figure S4 -Relevant part of the nucleotide sequence of the plasmid pSB2T-ccmk1$_{tsbp1}$-gfp. The DNA segment encompassing the *tac* promoter is written in red cases showing the so-called -35 (5'-TTGACA-3') and -10 (5'-TATAAT-3') promoter boxes, and the A transcription start site in upper cases. The Ccmk1$_{tsbp1}$ and **gfp** part of the ccmK1-fusion gene are colored in blue and green. GTTATC and GATAAC sequences are the scars corresponding to the cloning of the *Eco*RV DNA fragment harboring the Ccmk1$_{tsbp1}$-gfp fusion gene into the pSB2T plasmid opened at its unique *Hpa*I site. The cleavage site for *Hpa*I and *Eco*RV are respectively: ***Hpa*I, GTT/AAC** and ***Eco*RV, GAT/ATC**.

gatccgggcttatcgactgcacggtgcaccaatgcttctggcgtcaggcagccatcggaagctgtggtatggctgtgcaggtcgtaaatcactgcataattcgtgtcgct
caaggcgcactcccgttctggataatgttttttgcgccgacatcataacggttctggcaaatattctgaaatgagctg**TTGACA**attaatcatcggctcg**TATAAT**gtgt
gga**A**ttgtgagcggataacaatttcacacaggaaacagaattcccgggatctccgcg**GTTATC**gttgtcccgatggaggcttcatgaggcaatctctctactgtttttt
tgagaAGAGGAaacatct**ATG**GCAATTGCCGTCGGTATGATTGAAACCCTTGGTTTCCCCGCAGTGGTGGAAGCCGCAGACGCAATGGTGAAAGCTGCTCGCGTTACCCT
GGTAGGCTACGAGAAAATTGGGAGCGGTCGGGTCACCGTGATTGTCCGTGGCGATGTTTCTGAAGTGCAGGCTTCGGTTGCGGCGGGGGTCGAAAATGTGAAACGGGTGA
ATGGGGGTCAGGTGCTCTCCACCCACATCATTGCTCGTCCCCACGAAAACCTTGAGTACGTATTGCCCATTCGCTACACCGAAGCGGTGGAACAATTCCGCGAAAGCGTT
AGTGGTATCCGTCCGATGGGTCGCCCATCTAAAGGTGAAGAATTATTCACTGGTGTTGTCCCAATTTTGGTTGAATTAGATGGTGATGTTAATGGTCACAAATTTTCTGT
CTCCGGTGAAGGTGAAGGTGATGCTACTTACGGTAAATTGACCTTAAAATTTATTTGTACTACTGGTAAATTGCCAGTTCCATGGCCAACCTTAGTCACTACTTTCGGTT
ATGGTGTTCAATGTTTTGCGAGATACCCAGATCATATGAAACAACATGACTTTTTCAAGTCTGCCATGCCAGAAGGTTATGTTCAAGAAAGAACTATTTTTTTCAAAGAT
GACGGTAACTACAAGACCAGAGCTGAAGTCAAGTTTGAAGGTGATACCTTAGTTAATAGAATCGAATTAAAAGGTATTGATTTTAAAGAAGATGGTAACATTTTAGGTCA
CAAATTGGAATACAACTATAACTCTCACAATGTTTACATCATGGCTGACAAACAAAAGAATGGTATCAAAGTTAACTTCAAAATTAGACACAACATTGAAGATGGTTCTG
TTCAATTAGCTGACCATTATCAACAAAATACTCCAATTGGTGATGGTCCAGTCTTGTTACCAGACAACCATTACTTATCCACTCAATCTGCCTTATCCAAAGATCCAAAC
GAAAAGAGAGACCACATGGTCTTGTTAGAATTTGTTACTGCTGCTGGTATTACCCATGGTATGGATGAATTGTACAAA**CCC**GGGGATGTAGGG**TAA**taatgctggccatg
ggctagtgagtttgatagtttcataattgtccacggcagggtttaagccctgctttttgttgcccattggcaaactccaagacagccAAGCCGAATTCTGCA**GATAAC**

Amino-acids sequence of the Ccmk1$_{tsbp1}$-GFP fusion protein encoded by the pSB2T-ccmk1$_{tsbp1}$-gfp plasmid

MAIAVGMIETLGFPAVVEAADAMVKAARVTLVGYEKIGSGRVTVIVRGDVSEVQASVAAGVENVKRVNGGQVLSTHIIARPHENLEYVLPIRYTEAVEQFRESVSGIRPM
GRPSKGEELFTGVVPILVELDGDVNGHKFSVSGEGEGDATYGKLTLKFICTTGKLPVPWPTLVTTFGYGVQCFARYPDHMKQHDFFKSAMPEGYVQERTIFFKDDGNYKT
RAEVKFEGDTLVNRIELKGIDFKEDGNILGHKLEYNYNSHNVYIMADKQKNGIKVNFKIRHNIEDGSVQLADHYQQNTPIGDGPVLLPDNHYLSTQSALSKDPNEKRDHM
VLLEFVTAAGITHGMDELYKPGDVG*
